# Improving the Photocatalytic Reduction of CO_2_ to CO through Immobilisation of a Molecular Re Catalyst on TiO_2_

**DOI:** 10.1002/chem.201405041

**Published:** 2015-01-29

**Authors:** Christopher D Windle, Ernest Pastor, Anna Reynal, Adrian C Whitwood, Yana Vaynzof, James R Durrant, Robin N Perutz, Erwin Reisner

**Affiliations:** [a] Christian Doppler Laboratory for Sustainable SynGas Chemistry, Department of Chemistry, University of CambridgeLensfield Road, Cambridge CB2 1EW (U.K.); [b] Department of Chemistry, University of YorkHeslington, York YO10 5DD (U.K.); [c] Department of Chemistry, Imperial College LondonExhibition Road, London SW7 2AZ (U.K.); [d] Cavendish Laboratory, University of CambridgeCambridge, CB3 0HE, U.K.

**Keywords:** CO_2_ reduction, heterogeneous catalysis, immobilisation, photocatalysis, time-resolved spectroscopy

## Abstract

The photocatalytic activity of phosphonated Re complexes, [Re(2,2′-bipyridine-4,4′-bisphosphonic acid) (CO)_3_(L)] (ReP; L=3-picoline or bromide) immobilised on TiO_2_ nanoparticles is reported. The heterogenised Re catalyst on the semiconductor, ReP–TiO_2_ hybrid, displays an improvement in CO_2_ reduction photocatalysis. A high turnover number (TON) of 48 mol_CO_ mol_Re_^−1^ is observed in DMF with the electron donor triethanolamine at *λ*>420 nm. ReP–TiO_2_ compares favourably to previously reported homogeneous systems and is the highest TON reported to date for a CO_2_-reducing Re photocatalyst under visible light irradiation. Photocatalytic CO_2_ reduction is even observed with ReP–TiO_2_ at wavelengths of *λ*>495 nm. Infrared and X-ray photoelectron spectroscopies confirm that an intact ReP catalyst is present on the TiO_2_ surface before and during catalysis. Transient absorption spectroscopy suggests that the high activity upon heterogenisation is due to an increase in the lifetime of the immobilised anionic Re intermediate (*t*_50 %_>1 s for ReP–TiO_2_ compared with *t*_50 %_=60 ms for ReP in solution) and immobilisation might also reduce the formation of inactive Re dimers. This study demonstrates that the activity of a homogeneous photocatalyst can be improved through immobilisation on a metal oxide surface by favourably modifying its photochemical kinetics.

## Introduction

The efficient and selective reduction of CO_2_ is the key challenge to access carbon capture and utilisation (CCU) technologies. The green conversion of CO_2_ into the energy carrier CO is particularly attractive as it could be used, in combination with H_2_, as an important chemical feedstock to form syngas, and replace the steam reforming of fossil fuels in the petrochemical industry. Sustainable syngas provides a direct route to a greener chemical industry sector and the generation of transport fuel through Fischer–Tropsch chemistry.[1]

Photoelectrocatalytic CO_2_ reduction has been achieved on semiconductors,[2] such as p-type Si, CdTe, InP, GaAs, GaP, Cu_2_O[3] and n-type TiO_2_.[4] However, semiconductor materials often suffer from poor selectivity, producing a range of carbon-based products as well as H_2_. In contrast, enzymes display excellent selectivity and high turnover frequencies; a carbon monoxide dehydrogenase adsorbed on dye-sensitised TiO_2_ reduced CO_2_ selectively to CO with a turnover frequency (TOF) of 530 h^−1^.[5], [6] Major drawbacks of enzymes are their large size giving a correspondingly low ‘per volume’ activity, fragility and the restriction of their operation to specific temperatures, pH ranges and anaerobic atmospheres.

Synthetic molecular photo- and electrocatalysts can provide good stability combined with high product selectivity.[7] They may also be tuned by choosing from a range of metals and ligands.[8] For example, Fe porphyrin electrocatalysts have been reported to operate with high Faradaic efficiency and rate with overpotentials between 410 mV and 890 mV, depending on the molecular structure. Other examples are Re-, Ni-, Ru- and Pd-based catalysts, which operate under such overpotentials.[7,9] The photocatalytic reduction of CO_2_ using synthetic molecular catalysts can be achieved through two main strategies: They can receive electrons from an excited photosensitiser with appropriate energy requirements. Rhenium catalysts for CO_2_ reduction have been used in photocatalytic dyads of this type.[10] A second approach consists of using molecular photocatalysts that can perform both the functions of light harvesting and chemical catalysis.[11]

The rhenium tricarbonyl bipyridine complex with a defined leaving group L, [Re(bpy)(CO)_3_(L)], is one of very few mononuclear compounds that falls into this second category and acts as both a light absorber and a catalyst for the photoreduction of CO_2_ to CO.[11a–c] However, the two major drawbacks of such Re catalysts are their inefficient absorption of solar radiation and their low photostability and turnover numbers to generate CO (TON_CO_). The highest TON_CO_ reported to date for a Re complex acting as both light absorber and catalyst in solution in DMF/triethanolamine (TEOA) is 30, using *λ>*400 nm irradiation (L=Cl^−^).[11a, [12] A Re derivative with a thiocyanate leaving group gave a TON_CO_ of 26 (2 h, *λ>*400 nm)[8b] or a TON_CO_ of 30 (25 h, *λ*=365 nm).[13] A dinuclear system gave a TON_CO_ per Re centre of 30 after one hour of irradiation (*λ*=450 nm, LED).[14] Until recently, photocatalysis with Re complexes had only been performed in homogeneous solution. [Re(bpy)(CO)_3_L] complexes have been immobilised in various environments, but few of these systems have been reported as CO_2_ reduction photocatalysts.[15] Emerging heterogeneous systems[16] are advantageous as they facilitate catalyst recycling and increase the flexibility of solvents that may be used.[12,17] However, heterogenised Re photocatalysts have to date shown only low efficiency, with a maximum reported TON_CO_ of 7 in the absence of an additional dye.[12]

Herein we report two novel phosphonated Re bipyridine photocatalysts (ReP) for selective CO_2_ to CO reduction. Quantitative immobilisation of the ReP catalysts onto TiO_2_ nanoparticles results in an improvement of the TON_CO_ compared to previously reported homogeneous Re catalysts. To explain the nature of this enhanced photoactivity of ReP, the reaction mechanism and the kinetics of the catalytic intermediates were studied by time correlated–single photon counting and transient absorption spectroscopy. This spectroscopic study reveals an enhanced stability of the reaction intermediates when immobilising the ReP catalysts onto TiO_2_ and provides evidence for a reduction in the concentration of inactive Re dimers formed during catalysis, resulting in a 26-fold increase in the CO_2_ reduction yield for the heterogenised ReP on TiO_2_ compared to homogeneous ReP.

## Results and Discussion

### Synthesis and characterisation of ReP

**Figure 1:**
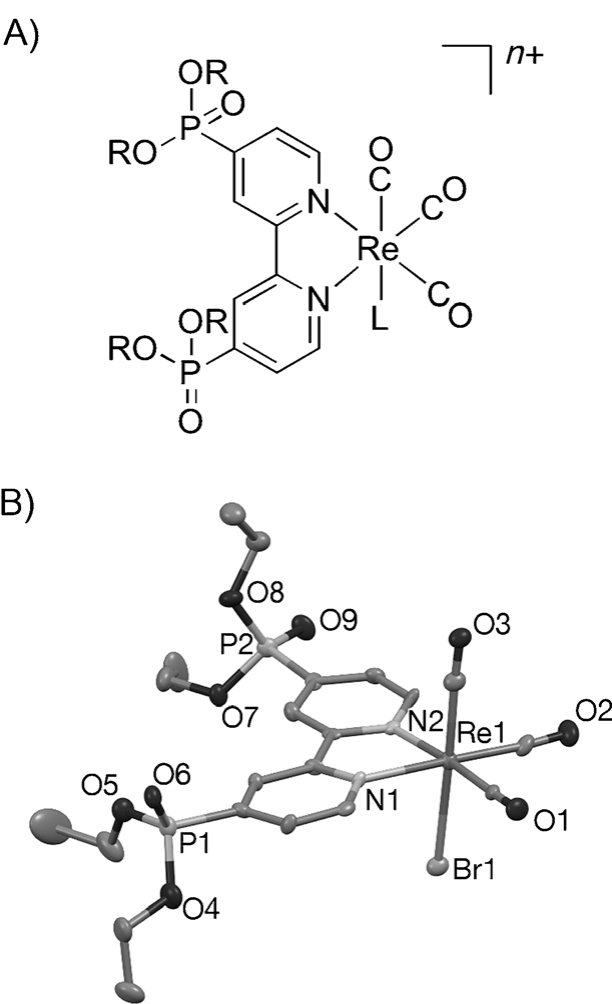
A) Structure of ReP catalysts. ^Et^ReP^Br^: R=Et, L=Br, *n*=0; ^Et^ReP^pic^: R=Et, L=3-picoline, *n*=1 (PF_6_^−^ counter-ion not shown); ReP^Br^: R=H, L=Br, *n*=0; ReP^pic^: R=H (isolated as neutral, mono-deprotonated species), L=3-picoline, *n*=0; B) ORTEP view of the molecular structure of ^Et^ReP^Br^ determined by X-ray diffraction with thermal ellipsoids set at 50 % probability. H atoms and atom-labelling for C atoms omitted for clarity.

Rhenium catalysts with phosphonated bipyridine ligands for immobilisation on metal oxides, three CO ligands in facial configuration and a 3-picoline (ReP^pic^) or bromide (ReP^Br^) leaving group were prepared (Figure 1). Heating [ReBr(CO)_5_] with tetraethyl 2,2′-bipyridine-4,4′-bisphosphonate in refluxing benzene gave ^Et^ReP^Br^ in 92 % yield. The bromide ligand in ^Et^ReP^Br^ was replaced upon heating with AgPF_6_ and 3-picoline in THF to yield ^Et^ReP^Pic^ in 77 %. The ethyl ester groups were dealkylated with ISiMe_3_ in CH_2_Cl_2_ followed by methanol treatment to give ReP^Pic^ in 39 % yield. ReP^Br^ was synthesised by heating [ReBr(CO)_5_] with 2,2′-bipyridine-4,4′-bisphosphonic acid in a refluxing toluene/methanol mixture in 44 % yield, or by reaction of ^Et^ReP^Br^ with BrSiMe_3_ in 62 % yield.

The composition and purity of the ReP complexes were confirmed by ^1^H, ^31^C and ^31^P NMR, UV/Vis and FT-IR spectroscopies, mass spectrometry and elemental analysis (see Experimental Section and Figures S1–S8 in the Supporting Information). Single crystals of ^Et^ReP^Br^ suitable for X-ray diffraction analysis were obtained from CH_2_Cl_2_/hexane and the crystal structure is shown in Figure 1 B. ^Et^ReP^Br^ displayed a Re1—N1 bond length of 2.163(7) Å, a Re1—Br1 bond length of 2.6291(10) Å and a torsion angle for NCCN in the 2,2′-bipyridine of 6.3(11)°. In comparison [ReCl(4,4′-dimethyl-2,2′-bipyridine)(CO)_3_] showed a Re—N bond length of 2.172 Å, a Re—Cl bond length of 2.489 Å and a torsion angle in the bipyridine of 3.01°.[8a] ReP^pic^ was detected by ESI-MS as a doubly deprotonated species in the negative ion mode and is likely to exist in a singly deprotonated form as a zwitterionic, overall charge-neutral complex in solution. The absence of signals for the PF_6_^−^ counter ion in the ^31^P NMR spectrum provided further evidence for a charge-neutral complex. Elemental analysis indicated that in ReP^Br^ the phosphonic acid groups are fully protonated and therefore that ReP^Br^ is also uncharged. ReP^pic^ and ReP^Br^ were both soluble in DMSO and H_2_O, but only ReP^Br^ in DMF.

### Assembly and characterisation of ReP–TiO_2_ hybrid

The quantitative immobilisation of ReP^pic^ on dispersed TiO_2_ nanoparticles (NPs; Evonik Aeroxide P25; 21 nm diameter) at sub-monolayer concentration was confirmed by UV/Vis spectrophotometry (*λ*=370 nm assigned to MLCT band in ReP^pic^). No ReP^pic^ remained in the supernatant after stirring ReP^pic^ (0.1 μmol) in aqueous solution with TiO_2_ (5 mg) for 1 h and centrifugation of the ReP^pic^-loaded TiO_2_ NPs (see the Supporting Information, Figure S9). ReP was loaded in water due to the insolubility of ReP^pic^ in most common organic solvents and to avoid adsorption of a carbon containing solvent on the TiO_2_ surface (to facilitate XPS analysis; see below). The maximum loading capacity was determined by UV/Vis spectrophotometry as 0.4 μmol per 5 mg TiO_2_, which is comparable to the loading capacity of phosphonated cobaloxime and ruthenium tris(2,2′-bipyridine) complexes on TiO_2_.[18]

**Figure 2:**
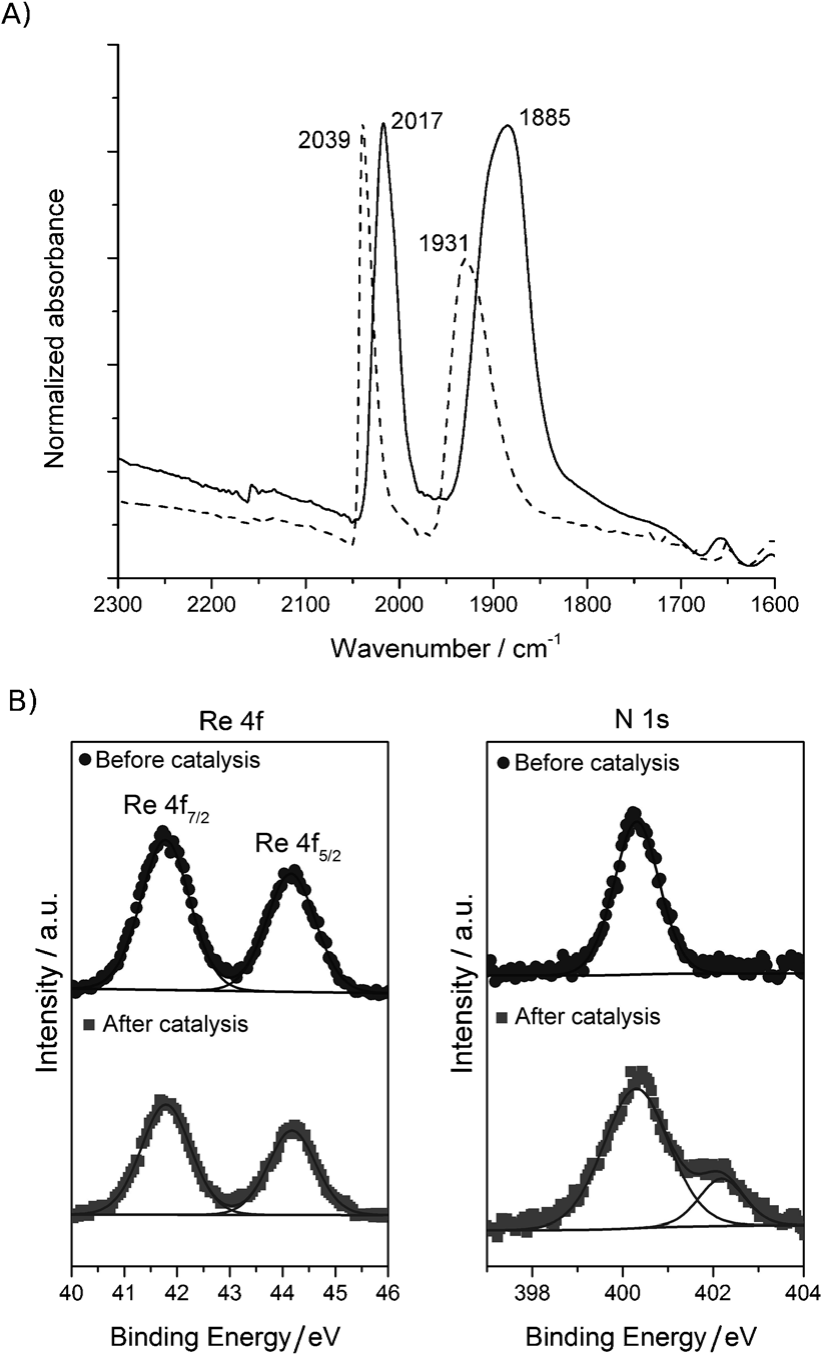
A) ATR-IR spectrum of ReP^pic^–TiO_2_ before and after 24 h of *λ>*420 nm irradiation in DMF/TEOA 5:1 under CO_2_. Spectrum after 24 h normalised to the maximum of the 0 h spectrum.; B) ReP^Br^–TiO_2_ XPS signals for Re before (circles, 1.08 %) and after (squares, 0.82 %) and for N before (circles, 2.74 %) and after (squares, 4.19 %) catalysis (2 h, *λ>*420 nm in 5:1 DMF/TEOA, under CO_2_).

Several techniques were subsequently used to characterise the ReP^pic^–TiO_2_ NP hybrid (Figure 2). An IR spectrum of ReP^pic^ in MeOH solution displayed CO stretching vibrations at 2037 and 1932 cm^−1^ (Table 1), whereas an attenuated total reflectance (ATR) spectrum gave signals at 2025 and 1890 cm^−1^. This difference in wavenumber is due to solid-state effects. Once loaded onto TiO_2_ but before exposure to DMF/TEOA, the ATR spectrum gives wavenumbers of 2039 and 1931 cm^−1^, which are very similar to the spectrum in MeOH solution. On the particle, the molecules of ReP^pic^ are dilute and experience no solid-state effects as they cannot interact with one another.

**Table 1:** Functional group IR signals (cm^−1^) for ^Et^ReP^Br^, ^Et^ReP^pic^ (CH_2_Cl_2_ solution) and ReP^pic^ (MeOH solution). n.d.=not determined.

^Et^ReP^Br^	^Et^ReP^pic^	ReP^pic^	ReP^pic^–TiO_2_	Assignment
**Solution**				
2024	2036	2037	n.d.	CO
1930	1927	1932	n.d.	CO
1901	—	—	n.d.	CO
				
**ATR**				
1258	1257	1153	1156	P—O
1011	1014	—	—	P—OEt
975	971	—	—	P—OEt

Signals corresponding to phosphorus–oxygen bond stretches can be observed in the ATR spectra (Table 1). There is little difference between the P—O and P—OEt stretches for ^Et^ReP^Br^ and ^Et^ReP^pic^ despite the difference in charge between the complexes. The P—O stretch in ReP^pic^ shows a low wavenumber shift of 100 cm^−1^ compared with ^Et^ReP^Br^ and ^Et^ReP^pic^. There is little difference between the P—O signal for unbound ReP^pic^ and that when bound to TiO_2_.

X-ray photoelectron spectroscopy (XPS) measurements of ReP^Br^–TiO_2_ confirmed the elemental composition upon immobilisation on TiO_2_. ReP^Br^ was utilised for XPS studies due to the additional element, which provides a distinct handle for the bromide (as leaving group) compared to the picoline complex. XPS measurements showed a composition of 1.08 % Re, 0.49 % Br, 2.74 % P and 2.52 % N, in good agreement with the expected 1:1 ratio for P to N and 2:1 for N to Re (Figure 2, as well as Figure S11 and Table S1 in the Supporting Information). The low Br percentage suggests that the Br is rapidly substituted in the aqueous solution.[19]

### Photocatalytic activity

**Figure 3:**
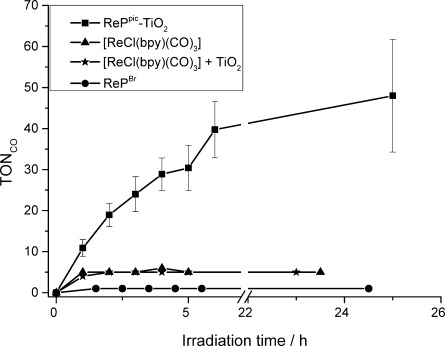
Photocatalytic CO production from CO_2_ under *λ>*420 nm irradiation with the ReP–TiO_2_ hybrid system compared to homogeneous [ReCl(bpy)(CO)_3_] with and without TiO_2_ and ReP^Br^ in solution (ReP^pic^ is insoluble in DMF). 0.1 μmol Re catalyst in 4.5 mL DMF/TEOA 5:1 was used with 5 mg TiO_2_. TON_CO_=mol_CO_ mol_Re_^−1^.

The ReP–TiO_2_ system displays CO_2_ photoreduction activity under UV-filtered simulated solar light (100 mW cm^−2^, *λ>*420 nm). Different amounts of ReP^pic^ on TiO_2_ in 5:1 DMF/TEOA (4.5 mL) were studied to identify optimal conditions for the heterogenised Re catalyst; different filters and metal oxide nanoparticles were also used. The turnover number (TON) is defined as molecules of CO produced per molecule of ReP catalyst after the system had stopped producing CO and is a measure of stability of the Re catalyst in the system. ReP^pic^ loadings of 0.05, 0.1, 0.2, 0.3, 0.4, 0.5 μmol per 5 mg TiO_2_ were tested and the highest TON (48) was achieved with a loading of 0.1 μmol ReP^pic^ on 5 mg TiO_2_ under *λ>*420 nm irradiation (see the Supporting Information, Table S2), which is hereafter denoted as the optimised system. ReP^Pic^–TiO_2_ gives a Re-based turnover frequency (TOF_CO_) of 8 h^−1^ during the first 3 h irradiation and a TON_CO_ of 48 after 1 day visible-light irradiation (Figure 3, Table 2). Control experiments, under comparable conditions but in the absence of either ReP^pic^, TEOA, TiO_2_, CO_2_ or light, did not generate significant amounts of CO (Table S2). For comparison, *λ>*420 nm irradiation of homogeneous solutions of [ReCl(bpy)(CO)_3_], ReP^Br^ and ^Et^ReP^pic^ (0.022 mm; 0.1 μmol of catalyst in 4.5 mL DMF/TEOA 5/1) resulted in final TON_CO_ values of 6, 2 and 0, respectively. The addition of 5 mg TiO_2_ did not alter the photoactivity of TiO_2_-anchor-free [ReCl(bpy)(CO)_3_] or that of ^Et^ReP^Br^, demonstrating the importance of immobilisation of ReP^pic^ on TiO_2_ for photoactivity. Replacing TiO_2_ with other metal oxides had a detrimental effect on the activity; *λ>*420 nm irradiation of ReP^pic^–SrTiO_3_ gave a TON of 20, whereas ReP^pic^–ZrO_2_ and ReP^pic^–ZnO gave TON of 8 and 10, respectively. ReP^pic^–CeO_2_ and ReP^pic^–ITO were photo-inactive (Table S3). The choice of metal oxide particle is therefore critical for the activity of immobilised ReP.

**Table 2:** TON_CO_ generated from photoreduction of CO_2_ with different catalytic systems and irradiation wavelength.

Catalytic System[a]	*λ* [nm]	TON_CO_±*σ*	Activity[b]
**Standard Conditions**			
ReP^pic^ (0.1 μmol), TiO_2_ (5 mg)	*>*420	48±14	0.96
ReP^Br^ (0.1 μmol), TiO_2_ (5 mg)	*>*420	52±12	1.04
[ReCl(bpy)(CO)_3_] (3.92 μmol)[c]	*>*420	30±3	N/A
[ReCl(bpy)(CO)_3_] (0.1 μmol)	*>*420	6±2	N/A
ReP^Br^ (0.1 μmol)	*>*420	2±1	N/A
**Different wavelengths**			
ReP^pic^ (0.1 μmol), TiO_2_ (5 mg)	*>*300	59±15	1.18
ReP^pic^ (0.1 μmol), TiO_2_ (5 mg)	*>*400	62±2	1.24
ReP^pic^ (0.1 μmol), TiO_2_ (5 mg)	*>*455	39±2	0.78
ReP^Br^ (0.1 μmol), TiO_2_ (5 mg)^[d]^	*>*495	16±4	0.32
[ReCl(bpy)(CO)_3_] (3.92 μmol)[c]	*>*455	33±10	N/A
[ReCl(bpy)(CO)_3_] (3.92 μmol)^[c,d]^	*>*495	9±4	N/A

[a] In 5:1 DMF/TEOA, 25 °C, irradiated for 1 day, TON_CO_=mol_CO_ mol_Re_^−1^;

[b] average mol_CO_ g
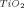
^−1^×10^−3^;

[c] optimised concentration for [ReCl(bpy)(CO)_3_];[11a] a TON of 6±2 was obtained with 0.1 μmol [ReCl(bpy)(CO)_3_] (*λ>*420 nm); [d] irradiated for 3 days.

Measurements with different neutral density filters and therefore reduced light intensity showed little effect on catalytic rate for ReP^pic^–TiO_2_ (0.1 μmol on 5 mg; see the Supporting Information, Figure S12), demonstrating that light harvesting was not performance-limiting under optimised conditions. Employing a 300, 400 or 420 nm cut-off filter resulted in comparable results (Table 2). Unexpectedly, a high TON_CO_ of 39 and 16 was observed with ReP–TiO_2_ with a *λ>*455 filter and a *λ>*495 nm filter, respectively (Table 2). To our knowledge, catalytic CO production by Re complexes with wavelengths as long as 495 nm has not been previously reported.

After 24 h of irradiating ReP^pic^–TiO_2_ (0.5 μmol on 5 mg), the nanoparticles were separated by centrifugation from the reaction solution, dried under vacuum and investigated with ATR-IR spectroscopy. Two metal carbonyl signals were clearly detected at *ν*=2017 and 1885 cm^−1^, indicating that a significant quantity of the Re catalyst remains intact on the TiO_2_ particles (Figure 2 A and Figures S13 and S14 in the Supporting Information). The FT-IR spectrum suggests that the [Re(bpy)(CO)_3_(L)] (L=labile ligand) symmetry is retained and vibrations display a substantial low frequency shift with respect to fresh ReP^pic^–TiO_2_, indicating that the Re centre has changed from cationic to neutral. This is due to coordination of an anionic species, probably deprotonated TEOA. The post-catalysis vibrations are comparable in wavenumber to those reported for a solution spectrum of the [Re(bpy)(CO)_3_(OCH_2_CH_2_N(CH_2_CH_2_OH)_2_)] complex (2006, 1897 and 1881 cm^−1^), but show only one low-wavenumber band.[20a] The CO stretches after catalysis are broader than beforehand, suggesting that they are probably compound peaks from multiple species. Proposed deactivation products for homogeneous Re systems include dimerisation during electrocatalysis[22] and formation of Re formate complexes.[23] It is likely that dimer formation would be less favourable due to immobilisation of the catalyst on TiO_2_.

The catalytic ReP^Br^–TiO_2_ system was characterised after 2 h of photocatalysis using XPS to investigate the stability of the molecular Re catalyst on the TiO_2_ surface (Figure 2 B, as well as Figure S15 and Table S1 in the Supporting Information). The percentage composition was Re 0.82 %, Br 0.42 %, P 1.99 % and N 4.19 %. The values for Re, Br and P are in a comparable ratio to the pre-catalysis measurements (see above), whereas the nitrogen content has increased. This observation can be explained by coordination of DMF and/or TEOA to the Re, as in known photocatalytic intermediates for comparable Re complexes.[20] In addition, the three hydroxy groups in TEOA can also bind to TiO_2_ and surface bound TEOA would result in a higher post-catalysis N content. The N 1 s signal at 400.3 eV, present before and after catalysis, has a very similar binding energy to signals previously assigned to the N in a Ru complex of 4,4′-dicarboxylic acid-2,2′-bipyridine adsorbed on TiO_2_ (400 eV).[21] The additional signal after catalysis at 402.2 eV is assigned to contributions from DMF and/or TEOA. The XPS results are consistent with FT-IR measurements, which indicate that a significant quantity of the molecular catalyst is present on the surface during catalysis.

### Spectroscopic characterisation

CO_2_ photoreduction is significantly more efficient for ReP catalysts anchored onto TiO_2_ compared with analogous concentrations in solution. ReP–TiO_2_ is also more efficient than an optimised homogeneous [ReCl(bpy)(CO)_3_] system (0.871 mm in DMF/TEOA) under simulated solar light.[11a] To understand the difference in efficiency between homogeneous and TiO_2_ immobilised catalysis, the ReP catalysts were investigated on TiO_2_ and in solution by time correlated–single photon counting (TC-SPC) and transient absorption spectroscopy (TAS). The catalytic mechanism of ReP for the reduction of CO_2_ was found to be independent of the nature of the labile ligand (Br or picoline).

The irradiation of [Re(bpy)(CO)_3_(L)] complexes (ReP) with visible light causes an MLCT transition generating a triplet state ^3^(ReP*) that emits at 600 nm with a characteristic broad signal (see the Supporting Information, Figure S16). ReP^pic^ displays a blue-shifted emission signal with respect to ReP^Br^. This feature of ReP^pic^ is characteristic of a cation, despite the zwitterionic feature of ReP^pic^ (Figure S16).[24] Both in solution and when immobilised onto TiO_2_, the luminescence of ReP decays within *t*_50 %_=20 ns. In good agreement with previous reports,[13,25] TEOA was observed to quench approximately 90 % of the emission within *t*_50 %_=10 ns due to the reductive electron transfer to ReP (see the Supporting Information, Figure S17). The luminescence intensity of ReP immobilised onto TiO_2_ is very similar to that of an analogous Al_2_O_3_ film functionalised with ReP (see the Supporting Information, Figure S18). Since Al_2_O_3_ is a semiconductor with a conduction band that does not allow for electron injection (−4.2 V vs. NHE),[26] these results indicate that electron injection from the photocatalyst to TiO_2_ does not take place.

Photoexcitation of homogeneous ReP^pic^ and ReP^pic^ immobilised on TiO_2_ (*λ*_ex_=415 nm) in the presence of TEOA results in two characteristic transient absorption features; a peak centred at *λ*_max_=500 nm and a broad transient signal in the near-IR region of the spectrum (800–900 nm). The latter band shows a smaller signal amplitude relative to the 500 nm peak, with this difference being more pronounced for ReP^pic^–TiO_2_ than for the homogeneous system (see the Supporting Information, Figure S19). These signals are not detected in the absence of an electron donor. As reported previously for analogous [Re(bpy)(CO)_3_(L)]-type catalysts, we have assigned the *λ*_max_=500 nm transient absorption peak to the reduced catalytic species ReP^−^, an important reaction intermediate in the CO_2_ photoreduction formed upon the reductive quenching of ReP* by TEOA along with the loss of the labile ligand (L).[25] This reaction intermediate was detected independently of the nature of the labile ligand (picoline or Br). It has previously been suggested that the catalytic mechanism of Re-based complexes involves the replacement of these ligands by DMF or TEOA.[20] However, the transient absorption in the near-IR has been previously assigned to the formation of catalytically inactive Re-dimers.[27] This assignment implies that the lower signal amplitude for ReP^pic^–TiO_2_ compared to homogeneous samples results from the formation of dimeric species, this being less favourable when the molecules are immobilised onto the surface of a metal oxide.

**Figure 4:**
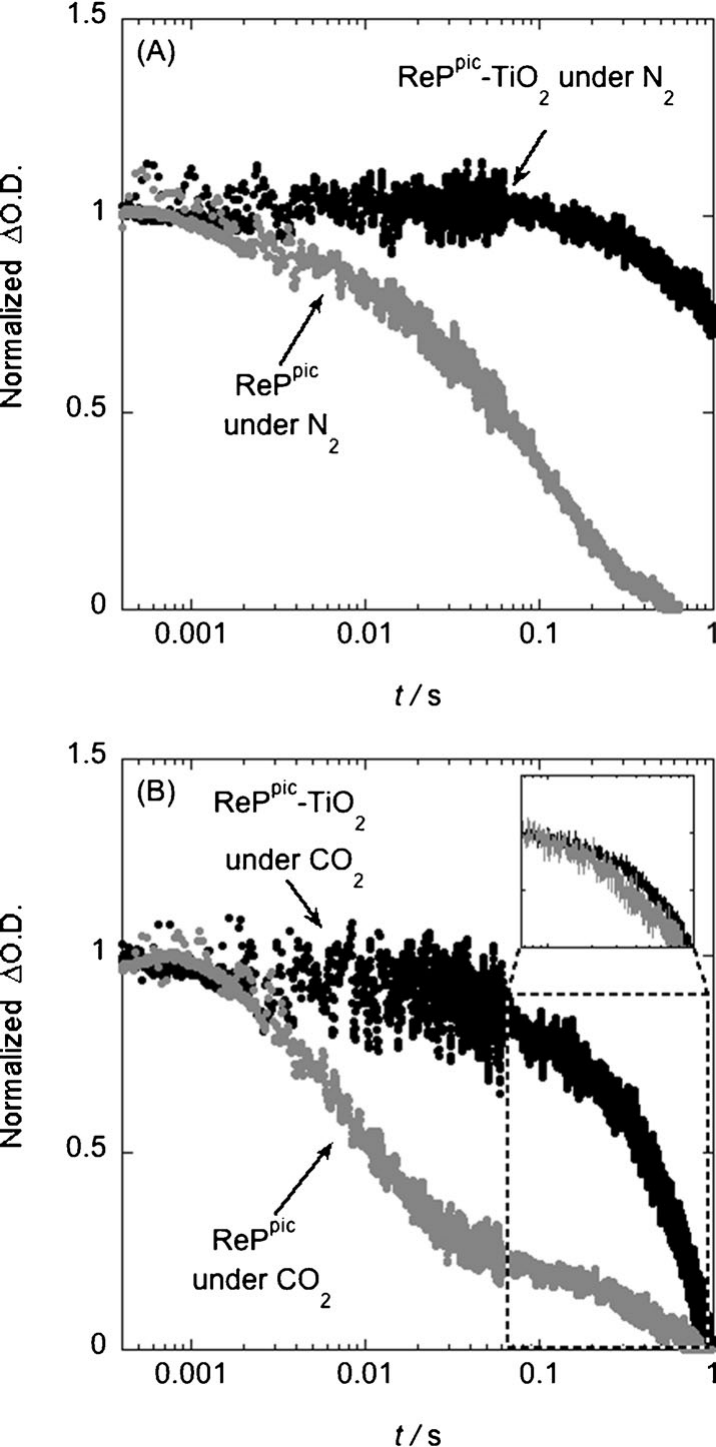
Transient absorption decays of the reduced intermediate ReP^−^ probed at 500 nm after photoexcitation of the catalyst with 415 nm light (ca. 300 μJ cm^−2^, 0.5 Hz repetition rate) in the presence of a sacrificial electron donor TEOA (1 m). A) ReP^pic^ in solution and anchored onto TiO_2_ under N_2_; B) ReP^pic^ in solution and anchored to TiO_2_ under CO_2_ with an inset showing a second normalisation of the kinetics of the slow phase for both systems.

The kinetics of ReP^pic^ in solution and anchored on TiO_2_ were also monitored by transient absorption spectroscopy in the millisecond to second timescales (Figure 4). The measurements were performed under either an N_2_ or a CO_2_ atmosphere, using TEOA (1 m in DMF) as sacrificial electron donor. In all cases, the decays were probed at 500 nm, corresponding to *λ*_max_ of the reduced catalytic intermediate ReP^−^. Under N_2_, the lifetime of ReP^−^ is more than one order of magnitude longer-lived when the catalyst is anchored onto TiO_2_ than in solution (*t*_50 %_>1 s for ReP^pic^–TiO_2_ and *t*_50 %_=60 ms for ReP^pic^ in homogeneous solution). The addition of CO_2_ in ReP^pic^–TiO_2_ samples shortens the lifetime of the transient absorption decay assigned to ReP^−^ to *t*_50 %_=400 ms. The decay of ReP^−^ in solution, in the presence of CO_2_, has a strong biphasic behaviour, with a fast component in the 1–10 ms timescale and a slow phase in the 100 ms–1 s timescale, indicative of a multiple step process. The effect of the lifetime of the reaction intermediates on the CO_2_ reduction photocatalysis is further discussed below.

The increase in lifetime of the reduced reaction intermediate observed when the catalyst is immobilised onto TiO_2_ suggests that the scaffolding provided by the metal oxide enhances the stability of this intermediate. Thus, the longer-lived catalytically active ReP^−^ species formed in ReP^pic^–TiO_2_ samples have a greater probability of encountering and consequently reacting with CO_2_ as well as of undergoing the second reduction necessary for the release of CO. This is consistent with our catalytic measurements showing a better performance of the ReP–TiO_2_ system. Our results are also in good agreement with a recent publication showing improved CO_2_ reduction yields in a TiO_2_ system loaded with a phosphonated Re catalyst and a molecular dye.[17] However, in this previous study the Re catalyst acted solely as an electrocatalyst with the dye acting as photosensitiser, whereas in our work ReP was employed as a photocatalyst without requiring an additional dye.

**Scheme 1:**
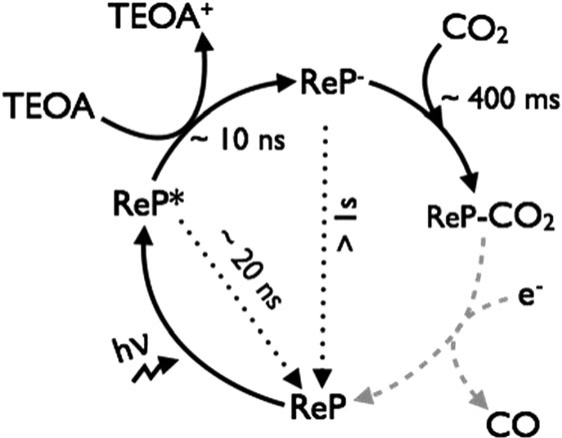
Proposed catalytic mechanism of a Re-based photocatalyst (ReP) immobilised on TiO_2_ for CO_2_ reduction under visible light illumination (solid lines). The deactivation pathways are represented with dotted lines. The second reduction step (dashed grey lines) was not observed by transient absorption spectroscopy measurements.

The transient absorption signal of ReP^pic^–TiO_2_ decays approximately one order of magnitude more rapidly when adding CO_2_, compared with the analogous system under N_2_. This faster decay can be most obviously assigned to the disappearance of ReP^−^ species upon reaction with CO_2_ in the 100 ms to 1 s timescale. Further evidence of the timescale of the interaction between ReP^−^ and CO_2_ is provided by comparing the kinetics of ReP^pic^ in solution and when immobilised on TiO_2_. From the biphasic transient absorption decay under CO_2_ of ReP^pic^ in solution, the kinetics of the slow phase are very similar to those of ReP^pic^–TiO_2_ (Figure 4 B, inset), suggesting that they involve the same process. Theoretical calculations supported the hypothesis that rhenium photocatalysts covalently attached to TiO_2_ show an optimum orientation for maximum reduction capacity.[15l] Thus, the lack of steric hindrance when immobilising ReP onto TiO_2_ suggests that the interaction of ReP^−^ with CO_2_ is expected to be comparable to a homogeneous system. We hypothesise that the fast phase of ReP^pic^ in solution is due to the formation of a reaction intermediate involved in the deactivation pathway of CO_2_ reduction, likely to correspond to the formation of the Re dimer.[22] The detailed nature of these catalytic and deactivation pathways are currently under investigation. We also note that the reduction of CO_2_ to CO requires two electron transfers. It has been previously hypothesised that this second reduction can take place through the interaction of two singly reduced ReP^−^ catalysts, or via the reaction of the carbon radical formed upon the deprotonation of [TEOA]^.+^ species with ReP^−^.[13], 20b, [25]] The kinetics of this second reduction, represented by dashed grey lines in Scheme 1, have not been observed in our transient absorption spectroscopy measurements. However, the higher CO_2_ reduction yields observed when ReP is immobilised onto TiO_2_ compared to homogeneous solution suggests that, in our system, the second electron transfer may occur through the sacrificial electron donor, as the immobilisation of ReP would hamper electron transfer between two Re molecules. The results of our kinetic studies are summarised in Scheme 1.

## Conclusions

We have synthesised two novel phosphonated rhenium bipyridine complexes, which bind quantitatively to TiO_2_ nanoparticles. The ReP–TiO_2_ hybrids can photoreduce CO_2_ to CO with high wavelength light (*λ>*495 nm), show good stability and show higher TONs than previously reported Re catalysts in the absence of dye as far as we are aware. The molecular structure is retained when the complex is immobilised onto the surface of the metal oxide before and during catalysis, although there are changes in the axial ligand, L. The unexpected enhancement in photocatalysis with ReP–TiO_2_ is explained by the significantly longer lifetime of the reduced catalytic Re species on TiO_2_, an important intermediate in the photoreduction mechanism, formed upon reductive quenching by the sacrificial electron donor. The near-IR region of the transient absorption spectra provide evidence that a reduced amount of inactive Re dimers is being formed in the catalytic cycle, which may also contribute to the higher activity of the ReP–TiO_2_ assembly. We have therefore shown that modification of the photochemical kinetics through photocatalyst immobilisation on metal oxide surfaces is a viable route to enhanced catalytic activity.

## Experimental Section

### Materials and Methods

Chemicals were obtained from the following suppliers: BrSiMe_3_, Et_3_N, triethanolamine, anhydrous DMF, ISiMe_3_, CeO_2_ particles (<25 nm)_,_ SrTiO_3_ particles (<100 nm), AgPF_6_, [Pd(PPh_3_)_4_], PPh_3_, P(OEt)_3_ (Aldrich); 3-picoline (BDH Chemicals); 4,4′-dibromo-2,2′-bipyridine (BOC Sciences); CO_2_/CH_4_ (BOC); silica 60 (Fluka); [Re_2_(CO)_10_] (Strem Chemicals). [ReBr(CO)_5_],[25] [ReCl(bpy)(CO)_3_],8a] tetraethyl 2,2′-bipyridine-4,4′-bisphosphonate[28] and 2,2′-bipyridine-4,4′-bisphosphonic acid[28] were prepared according to literature procedures. All solvents were dried before use in the synthetic part of the work. TiO_2_ nanoparticles (Aeroxide TiO_2_ P25 particles; anatase/rutile (8:2) mixture, average particle size 21 nm, BET 50 m^2^ g^−1^) and ZnO nanoparticles were a gift from Evonik Industries, ZrO_2_ nanoparticles were obtained from Skyspring Nanomaterials Inc. and CeO_2_, SrTiO_3_ and ITO from Sigma Aldrich.

### Physical measurements

^1^H and ^31^P NMR spectra were recorded either on a Bruker AVIII HD Smart Probe (400 MHz), a Bruker AVIII HD (400 MHz) or a Bruker ECS400 (400 MHz) spectrometer. ^1^H NMR spectra were referenced to the solvent residual peaks as an internal reference,[29] and ^31^P NMR spectra were referenced to an external standard (85 % H_3_PO_4_ in D_2_O). UV/Vis spectra were recorded on a Varian Cary 50 UV/Vis spectrophotometer using quartz glass cuvettes. Electrospray ionisation mass spectra (ESI-MS) were recorded on a Quattro LC spectrometer, Bruker Esquire 6000 or a Bruker microTOF instrument (for high-resolution (HR) ESI-MS measurements) and the theoretical and experimental isotope distributions were compared. *m*/*z* values are quoted for ^64^Zn, ^185^Re and ^79^Br. Elemental analysis was carried out by the microanalysis service of the Department of Chemistry, University of Cambridge. IR spectra were recorded on a Mattson RS FTIR instrument, averaging 64 scans at resolution 2 cm^−1^ or on a Thermo Scientific Nicolet iS50 FT-IR spectrometer. ATR spectra of the particles were recorded with an average of 256 scans. IR spectra of the KBr discs of the particles were taken with a PerkinElmer RX I instrument. ATR-IR spectra of particles before and after catalysis were run with 0.5 μmol ReP^pic^ on 5 mg TiO_2_ to obtain the strongest possible signals. X-ray photoelectron spectroscopy (XPS) measurements were carried out using an XR6 monochromated Al_Kα_ X-ray source (h*ν*=1486.6 eV) with a 900 mm spot size in an ultrahigh vacuum chamber (ESCALAB 250Xi). The pass energy was 20 eV. ReP^Br^ was chosen over ReP^pic^ (see below) for XPS due to the additional measurable element (Br). The sample after catalysis was washed with DMF, and dried under vacuum, followed by exposure to the XPS operating vacuum.

### Synthesis and Characterisation

**[ReBr(tetraethyl 2,2′-bipyridine-4,4′-bisphosphonate)(CO)_3_] (^Et^ReP^Br^)**: To a flame-dried two-neck 50 mL round bottom flask fitted with a reflux condenser was added tetraethyl 2,2′-bipyridine-4,4′-bisphosphonate (50 mg, 117 μmol) followed by [ReBr(CO)_5_] (47 mg, 117 μmol). The solids were subsequently suspended in anhydrous benzene (15 mL) and the reaction mixture heated to 65 °C. The reaction was monitored by IR spectroscopy (*ν*_CO_) showing completion after approximately 20 h. The solvent was removed under reduced pressure and the crude product redissolved in CH_2_Cl_2_ followed by precipitation of an orange solid upon addition of hexane. Yield: 84 mg, 92 %. Single crystals suitable for X-ray diffraction analysis were obtained by layering a CH_2_Cl_2_ solution of ^Et^ReP^Br^ with hexane. ^1^H NMR (400 MHz, CDCl_3_): *δ*=1.41 (td, 2.70 and 7.09 Hz, 12 H; CH_2_C*H*_3_), 4.26 (m, 8 H; C*H*_2_CH_3_), 7.84 (qd, 1.09, 5.41 Hz, 2 H; 5-bpy), 8.63 (d, 13.41 Hz, 2 H; 3-bpy), 9.19 ppm (t, 4.81 Hz, 2 H; 6-bpy); ^13^C{^1^H} NMR (100.6 MHz, CDCl_3_): *δ*=16.58 (CH_2_*C*H_3_), 63.88 (*C*H_2_CH_3_), 125.75 (bpy), 129.15 (bpy), 140.88 (bpy), 142.75 (bpy), 153.50 (bpy), 155.34 (CO), 196.21 ppm (CO); ^31^P{^1^H} NMR (109 MHz, CDCl_3_): *δ*=11.58 ppm (s); IR (CH_2_Cl_2_): 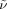
=2024 (s), 1930 (s), 1901 (s) cm^−1^ (*ν*(CO)); IR (ATR): 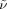
=2019 (*ν*(CO)), 1934 (*ν*(CO)), 1907 (*ν*(CO)), 1882, 1392 (P—O), 1258 (P—O), 1011 (P—OEt), 975 (P—OEt), 797 cm^−1^; HR-ESI-MS: *m*/*z* (%): 778.9705 (33; [*M*+H^+^]^+^, C_21_H_27_BrN_2_O_9_P_2_Re requires 778.9719); elemental analysis calcd (%) for C_21_H_26_BrN_2_O_9_P_2_Re: C 32.40, H 3.37, N 3.60, P 7.96; found: C 32.84, H 3.32, N 3.64, P 7.55.

**[Re(3-picoline)(tetraethyl 2,2′-bipyridine-4,4′-bisphosphonate) (CO)_3_][PF_6_] (^Et^ReP^pic^)**: A 250 mL two-neck round bottom flask was fitted with a reflux condenser and a gas valve and flame dried. AgPF_6_ (430 mg, 1.70 mmol) was added under Ar followed by THF (60 mL) and 3-picoline (3 mL). ^Et^ReP^Br^ (600 mg, 771 μmol) was added and the solution refluxed until the reaction was completed after approximately 20 h. The reaction was monitored by IR spectroscopy (*ν*_CO_). The solution was filtered and the filtrate evaporated to dryness. The oil residue was dried under vacuum overnight and then redissolved in CH_2_Cl_2_. The product was precipitated with hexane, collected by filtration and washed exhaustively with hexane. The product was purified by column chromatography (SiO_2_ with EtOAc and CH_2_Cl_2_/CH_3_OH (95:5) and Sephadex LH20 with THF and CH_3_OH). Yield: 553 mg, 77 %. ^1^H NMR (400 MHz, CDCl_3_): *δ*=1.35 (m, 12 H; CH_2_C*H*_3_), 2.25 (s, 3 H; picoline-CH_3_), 4.22 (m, 8 H; C*H*_2_CH_3_), 7.35 (m, 1 H; picoline-5), 7.60 (d, 7.81 Hz, 1 H; picoline-4), 8.00 (d, 5.47 Hz, 1 H; picoline-6), 8.10 (s, 1 H; picoline-2), 8.14 (q, 5.48 Hz, 2 H; bpy-5), 8.60 (d, 13.31 Hz, 2 H; bpy-3), 9.38 ppm (t, 4.99 Hz, 2 H; bpy-6);^13^C{^1^H} NMR (100.6 MHz, CDCl_3_): *δ*=16.31 (CH_2_C*H*_3_), 18.18 (picoline CH_3_), 64.16 (C*H*_2_CH_3_), 126.41 (bpy-3), 126.84 (picoline-5); 131.12 (bpy-5), 137.74 (picoline-3), 140.74 (picoline-4), 142.34 (bpy-4), 144.21 (bpy-2), 148.99 (picoline-6), 151.72 (picoline-2), 154.17 (bpy-6), 155.21 (CO), 190.02 (CO), 194.53 ppm (CO); ^31^P NMR (109 MHz, CDCl_3_): *δ*=10.59 (s, PO_3_Et_2_), −143.87 ppm (sep, 720 Hz, PF_6_); IR (CH_2_Cl_2_): 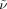
=2036 (s), 1927 cm^−1^ (s, broad; *ν*(CO)); IR (ATR): 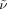
=2031 (*ν*(CO)), 1910 (*ν*(CO)), 1396, 1257 (P—O), 1014 (P—OEt), 971 (P—OEt), 833 cm^−1^; HR-ESI-MS: *m*/*z* (%): 790.1233 (59; *M*^+^, C_27_H_33_N_3_O_9_P_2_Re^+^ requires 790.1216).

**[Re(3-picoline)(2,2′-bipyridine-4-phosphonic acid-4′-hydroxyoxidophosphoryl)(CO)_3_] (ReP^pic^)**: A Schlenk tube was charged with ^Et^ReP^pic^ (179 mg, 191 μmol) and dried under reduced pressure at 40 °C overnight, followed by cooling to 0 °C. A solution of ISiMe_3_ in CHCl_3_ (1 m) was prepared in the glovebox, cooled to 0 °C and added by cannula (1.09 mL, 7.64 mmol of ISiMe_3_) to the reaction mixture. The red solution was stirred at 0 °C for 5 min and then evaporated to dryness and dried under vacuum for 72 h. The solid was redissolved in dry chloroform (1 mL), followed by the addition of methanol (1 mL). The solution was stirred at 0 °C for 10 min, allowed to warm to room temperature under stirring for 3 h and then evaporated to dryness and dried under vacuum for 24 h. The solid was precipitated from methanol with diethyl ether. Yield: 50 mg, 39 %. ^1^H NMR (400 MHz, CD_3_OD): *δ*=2.22 (s, 3 H; picoline-CH_3_), 7.25 (dd, 5.71 and 8.08 Hz, 1 H; picoline), 7.75 (d, 8.04 Hz, 1 H; picoline), 8.12 (q, 5.44 Hz, 2 H; bpy), 8.17 (d, 5.55 Hz, 1 H; picoline), 8.25 (s, 1 H; picoline), 8.77 (d, 12.70 Hz, 2 H, bpy), 9.44 ppm (dd, 3.22, 5.63 Hz, 2 H; bpy); ^31^P{^1^H} NMR (109 MHz, CD_3_OD): *δ*=6.68 ppm (s); IR (ATR): 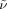
=2025 (s), 1890 (s, broad; *ν*(CO)), 1153 cm^−1^; IR (CH_3_OH): 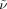
=2037 (s), 1932 cm^−1^ (s, broad; *ν*(CO)); ESI-MS (negative): *m*/*z*(%): 676.4 (48; [*M*^+^−2 H^+^]^−^, C_19_H_15_N_3_O_9_P_2_Re requires 676.0), 583.4 ([*M*^+^−2 H^+^−C_6_H_7_N]^−^, C_13_H_8_N_2_O_9_P_2_Re requires 582.9).

**[ReBr(2,2′-bipyridine-4,4′-bisphosphonic acid)(CO)_3_] (ReP^Br^)**:

**Method 1**: A Teflon capped high-pressure vessel was charged with [ReBr(CO)_5_] (316 mg, 778 μmol), 2,2′-bipyridine-4,4′-bisphosphonic acid (246 mg, 778 μmol), toluene (20 mL) and methanol (3 mL), and the reaction mixture was refluxed overnight. The tube was allowed to cool and the solids collected by filtration. These were redissolved in methanol and diethyl ether added to precipitate a yellow solid. The suspension was filtered to yield an orange solution and the solvent was removed under reduced pressure to yield an orange solid. Yield: 227 mg, 44 %. ^1^H NMR (400 MHz, CD_3_OD): *δ*=7.99 (dd, 3.91, 5.96 Hz, 2 H; bpy); 8.77 (d, 13.45 Hz, 2 H; bpy); 9.22 ppm (dd, 3.91, 5.96 Hz, 2 H; bpy); ^31^P{^1^H} NMR (162 MHz, CD_3_OD): *δ*=7.82 ppm (s); IR (ATR): 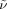
=2024 (s, (*ν*(CO)), 1882 (s, broad, (*ν*(CO)), 1606, 1476, 1395, 1155 (*ν*(P—O)), 1000, 935, 841 cm^−1^; IR (CH_3_CH_2_OH): 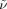
=2026 (s), 1929 (s), 1911 cm^−1^ (s; *ν*(CO)); elemental analysis calcd (%) for C_13_H_10_BrN_2_O_9_P_2_Re: C 23.43, H 1.51, N 4.20, P 9.30; found: C 23.54, H 1.75, N 4.01, P 9.23.

**Method 2**: A Schlenk tube was charged with ^Et^ReP^Br^ (100 mg, 128 μmol) and dried under vacuum overnight. Anhydrous CHCl_3_ (5 mL) and then BrSiMe_3_ were added (1.2 mL, 9.09 mmol) to the Schlenk tube. The tube was refluxed for 24 h and the progress of the reaction monitored by ^31^P NMR spectroscopy (appearance of a peak at −7.16 ppm in the CHCl_3_ and BrSiMe_3_ reaction solution). The solution was then evaporated to dryness and dry MeOH (3 mL) added. The solution was stirred for 3 h, evaporated to dryness and dried overnight under high vacuum at room temperature. The product was purified by precipitation with diethyl ether from a saturated methanol solution. Yield: 53 mg, 62 %. ^1^H NMR (400 MHz, [D_6_]DMSO): *δ*=7.92 (dq, 0.64, 5.55 Hz, 2 H; bpy), 8.79 (d, 13.51 Hz, 2 H; bpy), 9.16 ppm (dd, 0.66, 5.73 Hz, 2 H; bpy); ^31^P{^1^H} NMR (162 MHz, [D_6_]DMSO): *δ*=10.85 ppm (s); IR (CH_3_OH): 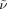
=2027 (s), 1928 (s), 1912 cm^−1^ (s; *ν*(CO)); HR-ESI-MS (negative): *m*/*z* (%): 582.9248 ([*M*−2 H^+^−Br^−^]^−^, C_13_H_8_N_2_O_9_P_2_Re requires 582.9235).

### Single crystal X-ray diffraction studies

Diffraction data were collected at 110 K on an Oxford Diffraction SuperNova diffractometer with MoK_α_ radiation (*λ*=0.71073 Å) using a EOS CCD camera. The crystal of ^Et^ReP^Br^ was cooled with an Oxford Instruments Cryojet. Diffractometer control, data collection, initial unit cell determination, frame integration and unit-cell refinement was carried out with “CrysAlisPro”.[30] Face-indexed absorption corrections were applied using spherical harmonics, implemented in SCALE3 ABSPACK scaling algorithm.[31] OLEX2[32] was used for overall structure solution, refinement and preparation of computer graphics and publication data. Within OLEX2, the algorithm used for structure solution was “charge-flipping” using the superflip program.[33] Refinement by full-matrix least-squares used the SHELXL-97[34] algorithm within OLEX2.[32] All non-hydrogen atoms were refined anisotropically. Hydrogen atoms were placed using a “riding model” and included in the refinement at calculated positions. Crystal data and structure refinement details (see the Supporting Information, Table S4), selected bond distances and angles (Table S5) and details about the X-ray crystal structure refinement are given in the Supporting Information.

### Assembly of colloidal ReP–TiO_2_ hybrid system

ReP was pre-loaded onto the TiO_2_ nanoparticles by stirring ReP with dispersed TiO_2_ in H_2_O for one hour protected from light. The particles were separated by centrifugation and removal of the supernatant, followed by drying under high vacuum at room temperature overnight. In a typical procedure, a solution of ReP^pic^ (1.36 mg) in H_2_O (10 mL) was added dropwise to a sonicated suspension of TiO_2_ (100 mg) in H_2_O (20 mL) with stirring. The mixture was stirred for 1 h, centrifuged, the supernatant removed and the ReP^pic^-modified TiO_2_ powder dried by high vacuum at room temperature overnight.

### Photocatalytic studies

A reaction mixture of DMF/TEOA 5:1 (4.5 mL) and the catalyst were added to the light-protected photoreactor, which was subsequently sealed with a rubber septum and air was replaced by bubbling through a needle with CO_2_ containing 2 % CH_4_ (internal GC standard) before irradiation. Photocatalytic experiments were performed with a Solar Light Simulator (Newport Oriel, 100 mW cm^−2^) equipped with an air mass 1.5 global filter (AM 1.5G). The irradiation wavelengths were controlled using UV and blue-light cut off-filters (UQG optics) and IR irradiation was filtered by a water filter (path length 10 cm). The temperature of the photoreactor was kept constant at 25 °C in all experiments with a water circulator connected to a water-jacketed reservoir. The irradiated cross section of the solution in the vials was approximately 6.3 cm^2^. The photoreactors consisted of glass tubes with volumes between 14.2 and 14.5 mL with corresponding headspace volumes of 9.7 and 10 mL, respectively. Periodic headspace gas analysis (30 μL) allowed quantification of CO at regular time intervals with an Agilent 7800 Series gas chromatograph (GC) equipped with a HP-PLOT/Q column (0.53 mm diameter) attached to a HP-5 column (0.32 mm diameter). The GC oven temperature was kept constant at 45 °C, He was used as carrier gas at an approximate flow rate of 2 mL min^−1^ and a thermal conductivity detector (TCD) was used. TON_CO_ assumes quantitative binding of ReP^pic^ to the particle and is given as the average over a minimum of three runs. The errors are given as ±1 standard deviation (*σ*).

### Spectroscopic measurements

Homogeneous samples for spectroscopic measurements were prepared by dissolving the photocatalyst (0.1 mm) in a water/DMF mixture (1:2). When required, TEOA was added to the solution to give a concentration of 1 m. Anatase TiO_2_ films were employed for spectroscopic measurements, because their smaller particle size and decreased scattering effect offer an enhanced optical transparency compared to that of P25 analogues. Anatase TiO_2_ films (8 μm thick, area 1 cm×1.5 cm) were prepared from a colloidal paste as reported previously and Al_2_O_3_ was prepared as reported previously,[35] and deposited onto microscopic slides by Doctor blading. The films were functionalised by dipping the TiO_2_ slides into a 0.3 mm aqueous solution of ReP during 48 h in the dark, to avoid degradation of the catalyst. The samples were then dried under vacuum for approximately 1 h to remove any traces of water. The samples were kept in the dark before and in between the measurements.

The millisecond to second transient absorption measurements were performed using a home built system as reported previously.[36] The samples were excited with a 415 nm laser pulse (∼300 μJ cm^−2^, 0.5 Hz rep. rate, 6 ns pulse width) and, unless otherwise stated, transient absorption decays were monitored at 500 nm. The decays observed were the average between 500 and 1000 laser pulses. N_2_ was used to purge the samples (>15 min) before each measurement, unless otherwise stated.

Time correlated–single photon counting measurements were performed using a Horiba Jobin Yvon TBX Fluorocube system. As excitation source, a pulsed laser with 404 nm nominal wavelength at a repetition rate of 100 kHz was used. The photoluminescence of a 0.1 mm ReP solution in N_2_-purged water, and a ReP–TiO_2_ film in air was monitored at 600 nm for a fixed period of time (300 s). The instrument response was measured at the full width half maximum, showing typically a 200–250 ps value.
