# Supplementary material for: Improving the Photocatalytic Reduction of CO2 to CO through Immobilisation of a Molecular Re Catalyst on TiO2
Source: Chemistry. 2015 Jan 29;21(9):3746–54. doi: 10.1002/chem.201405041 (PMC4471553; doi:10.1002/chem.201405041)
Supplement: Supplementary file 1 [file chem0021-3746-sd1.pdf]

CHEMISTRY
A European Journal

Supporting Information

© Copyright Wiley-VCH Verlag GmbH & Co. KGaA, 69451 Weinheim, 2015

Improving the Photocatalytic Reduction of CO_2 to CO through Immobilisation of a Molecular Re Catalyst on TiO_2

Christopher D. Windle,[a, b] Ernest Pastor,[c] Anna Reynal,*[c] Adrian C. Whitwood,[b] Yana Vaynzof,[d] James R. Durrant,[c] Robin N. Perutz,*[b] and Erwin Reisner*[a]

chem_201405041_sm_miscellaneous_information.pdf

Supporting Information

Contents

Experimental Section	page S2
Supporting Tables S1–S5	page S2
Supporting Figures S1–S19	page S6

Experimental Section.

Details about X-ray crystal structure refinement. The crystal showed evidence of non-merohedral twinning [with the worst bad reflections $(F_c^2-F_o^2)$/esd all negative] but there was too much overlap to allow separation using CrysAlis. There is a significant residual density peak between the carbon and oxygen of the CO trans to the bromide. This may be due to a minor form with the CO and Br sites exchanged, however attempts to model this failed to give a satisfactory result.

Supporting Tables.

Table S1. Elemental percentages derived from the X-ray photoelectron spectra for ReP^{Br}-TiO_2 before and after 2 h photocatalysis (DMF:TEOA 5:1, λ >420 nm, under CO_2). C, O, Ti and Sn were also found, originating from the substrate (TiO_2 on fluorine doped tin oxide coated glass), C and O from the catalyst and from surface contamination.

Element	Before catalysis / %	After catalysis / %
Re	1.08	0.82
Br	0.49	0.42
P	2.74	1.99
N	2.52	4.19

Table S2. Photocatalytic CO_2 to CO conversion with ReP-TiO_2 under different conditions (optimization and control experiments).

#	Catalytic Nanoparticle System	Experimental Conditions	$TON_{CO} \pm \sigma$
Different loadings of ReP on TiO_2			
10	ReP^{pic} (0.05 µmol), TiO_2 (5 mg)	TEOA, visible light (λ >420 nm, 100 mW cm^{-2})	29±13
11	ReP^{pic} (0.2 µmol), TiO_2 (5 mg)	TEOA, visible light (λ >420 nm, 100 mW cm^{-2})	31±13
12	ReP^{pic} (0.3 µmol), TiO_2 (5 mg)	TEOA, visible light (λ >420 nm, 100 mW cm^{-2})	23±8
13	ReP^{pic} (0.4 µmol), TiO_2 (5 mg)	TEOA, visible light (λ >420 nm, 100 mW cm^{-2})	11±8
14	ReP^{pic} (0.5 µmol), TiO_2 (5 mg)	TEOA, visible light (λ >420 nm, 100 mW cm^{-2})	18±2
Control experiments			
15	No Re, TiO_2 (5 mg)	TEOA, visible light (λ >420 nm, 100 mW cm^{-2})	0±0
16	ReP^{pic} (0.1 µmol), TiO_2 (5 mg)	no TEOA, visible light (λ >420 nm, 100 mW cm^{-2})	3±6
17	ReP^{pic} (0.1 µmol), TiO_2 (5 mg)	Under N_2, TEOA, visible light (λ >420 nm, 100 mW cm^{-2})	0±0
18	ReP^{Br} (0.1 µmol), no TiO_2	TEOA, visible light (λ >420 nm, 100 mW cm^{-2})	2±1
19	[ReCl(bpy)$(CO)_3$] (0.1 µmol), TiO_2 (5 mg)	TEOA, visible light (λ >420 nm, 100 mW cm^{-2})	8±3
20	[ReCl(bpy)$(CO)_3$] (0.1 µmol), TiO_2 (5 mg)	TEOA, (λ >455 nm, 100 mW cm^{-2})	3±1
21	[ReCl(bpy)$(CO)_3$] (0.1 µmol)	TEOA, visible light (λ >420 nm, 100 mW cm^{-2})	6±2
22	$^{Et}ReP^{Br}$ (0.1 µmol), TiO_2 (5 mg)	TEOA, visible light (λ >420 nm, 100 mW cm^{-2})	0±0

Table S3. Photocatalytic CO_2 to CO conversion with different ReP-nanoparticle hybrids.

#	Catalytic Nanoparticle System[a]	Experimental Conditions	$TON_{CO} \pm \sigma$
ReP with different metal oxide nanoparticles			
23	ReP^{pic} (0.1 μmol), ZrO_2 (5 mg)	TEOA, visible light (λ >420 nm, 100 mW cm^{-2})	8±2
24	ReP^{pic} (0.1 μmol), $SrTiO_3$ (5 mg)	TEOA, visible light (λ >420 nm, 100 mW cm^{-2})	20±6
25	ReP^{pic} (0.1 μmol), CeO_2 (5 mg)	TEOA, visible light (λ >420 nm, 100 mW cm^{-2})	0±0
26	ReP^{pic} (0.1 μmol), ITO (5 mg)	TEOA, visible light (λ >420 nm, 100 mW cm^{-2})	0±0
27	ReP^{pic} (0.1 μmol), ZnO (5 mg)	TEOA, visible light (λ >420 nm, 100 mW cm^{-2})	10±3

a) BET surface area of commercial metal oxides: $SrTiO_3$ 10 m^2 g^{-1}, CeO_2 23.3 m^2 g^{-1}, ITO 27 m^2 g^{-1}, ZnO 20-25 m^2 g^{-1}

Table S4. Single Crystal Data and Details of Data Collection for $^{Et}ReP^{Br}$

chemical formula	$C_{21}H_{26}BrN_2O_9P_2Re$	crystal size (mm)	0.11 × 0.06 × 0.02
M_r (g mol^{-1})	778.49	color, shape	Orange, needles
crystal system	monoclinic	T (K)	110.05(10)
space group	$P2_1/c$	μ (Mo K, mm^{-1})	0.7107
a (Å)	6.24242(19)	2Θ range, deg	6.62 to 52.04
b (Å)	34.8965(11)	total no. of data	9824
c (Å)	12.2985(4)	no. of unique data	5187
α (deg)	90.00	No. of parameters	317
β (deg)	99.287(3)	R_1 [I>2σ (I)]	0.0678
γ (deg)	90.00	wR_2	0.1124
V (Å^3)	2643.98(14)	R_1 [all data]	0.0816
Z	4	wR_2	0.1170
ρ_{calc}	1.956	GOF	1.310

Table S5. Selected Bond Distances (Å) and Angles (deg) for $^{Et}ReP^{Br}$.

Re1–N1	2.163(7)	N2–Re1–N1	74.3(3)
Re1–Br1	2.6291(10)	C2–Re1–C1	87.3(4)
Re1–C1	1.939(10)	C1–Re1–C3	89.7(4)
P1–C6	1.800(9)	C7–C8–C9–C10	4.4(14)

Supporting Figures.

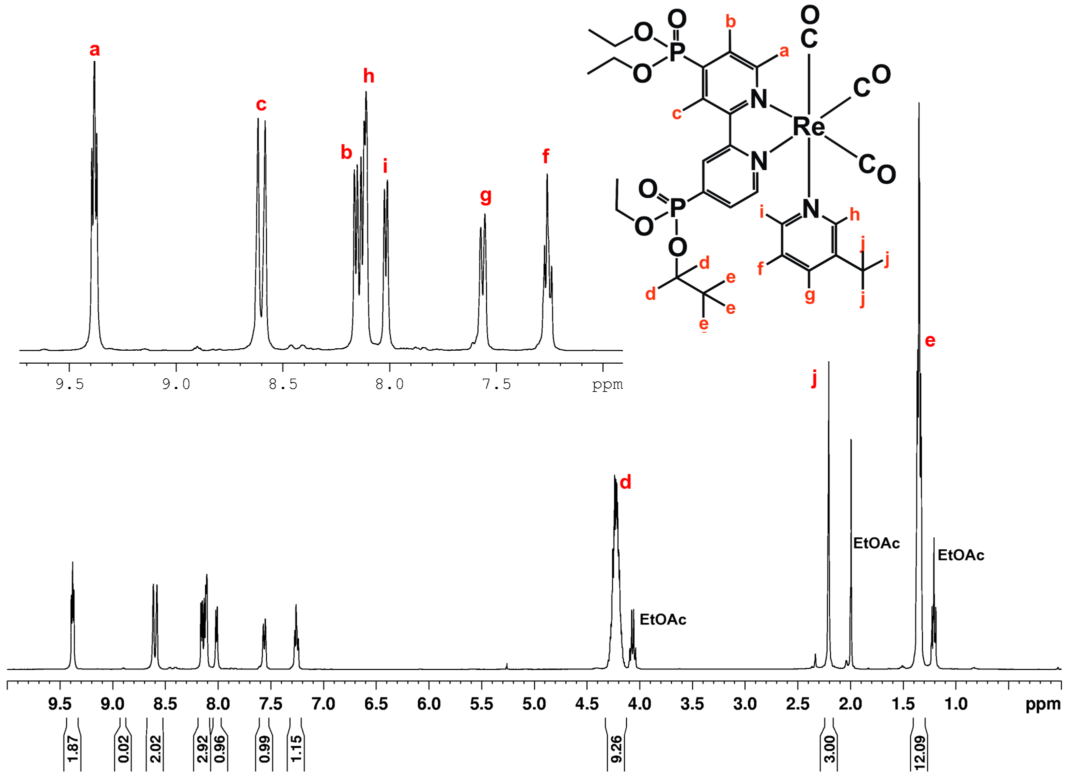

Figure S1. ^{1}H NMR spectrum of ^{Et}ReP^{pic} with structural assignment (CDCl$_3$, 400 MHz)

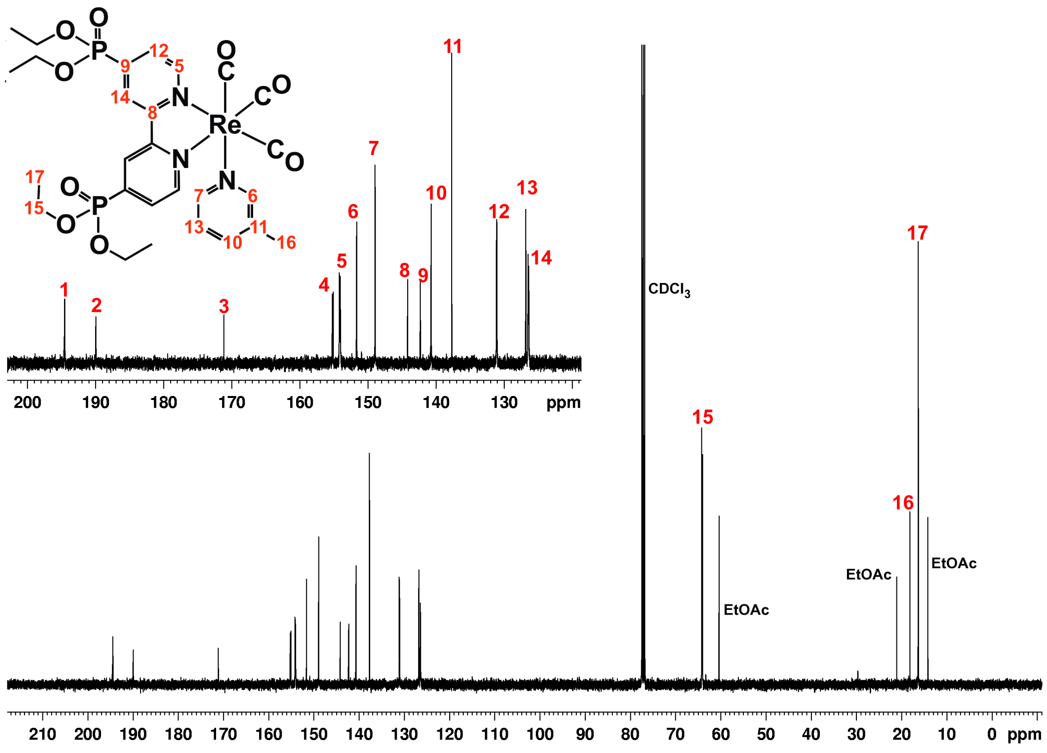

Figure S2. ^{13}C{^{1}H} NMR spectrum of ^{Et}ReP^{pic} with structural assignment (CDCl$_3$, 100.6 MHz)

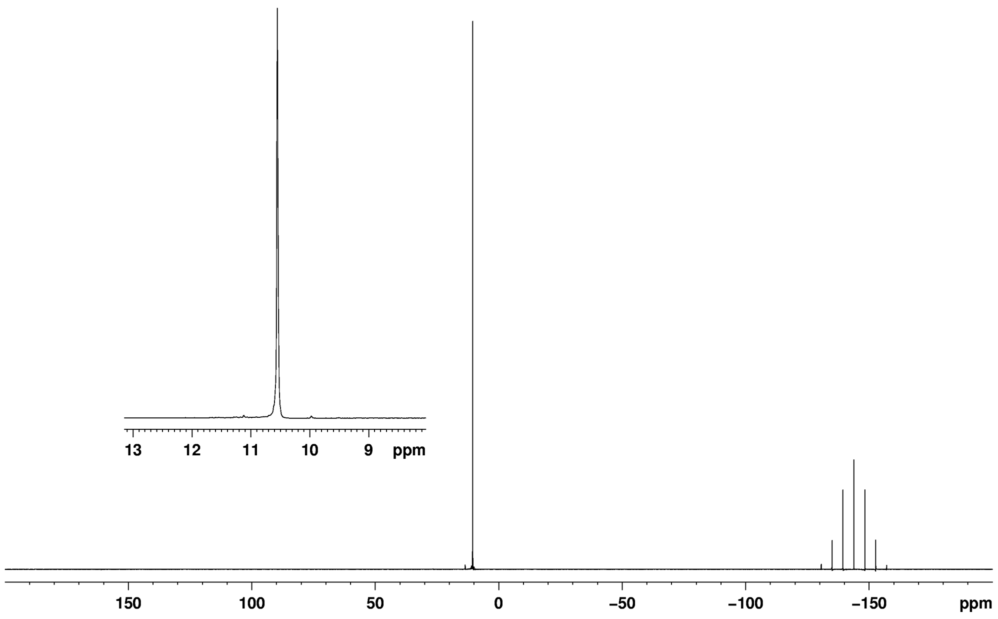

Figure S3. $^{31}P\{^{1}H\}$ NMR spectrum of $^{Et}ReP^{pic}$ ($CDCl_3$, 109 MHz).

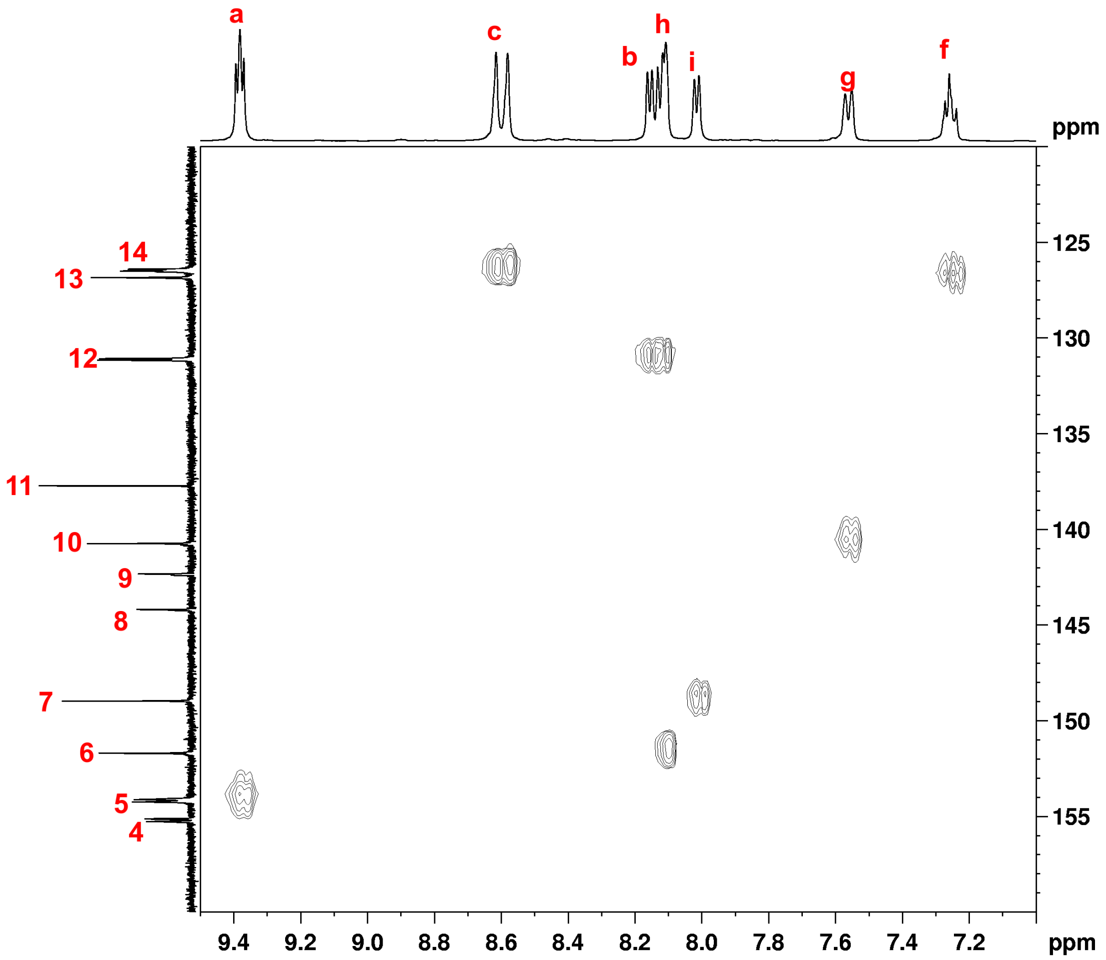

Figure S4. ^{1}H-^{13}C HMQC NMR spectrum of $^{Et}ReP^{pic}$ ($CDCl_3$, 400 MHz).

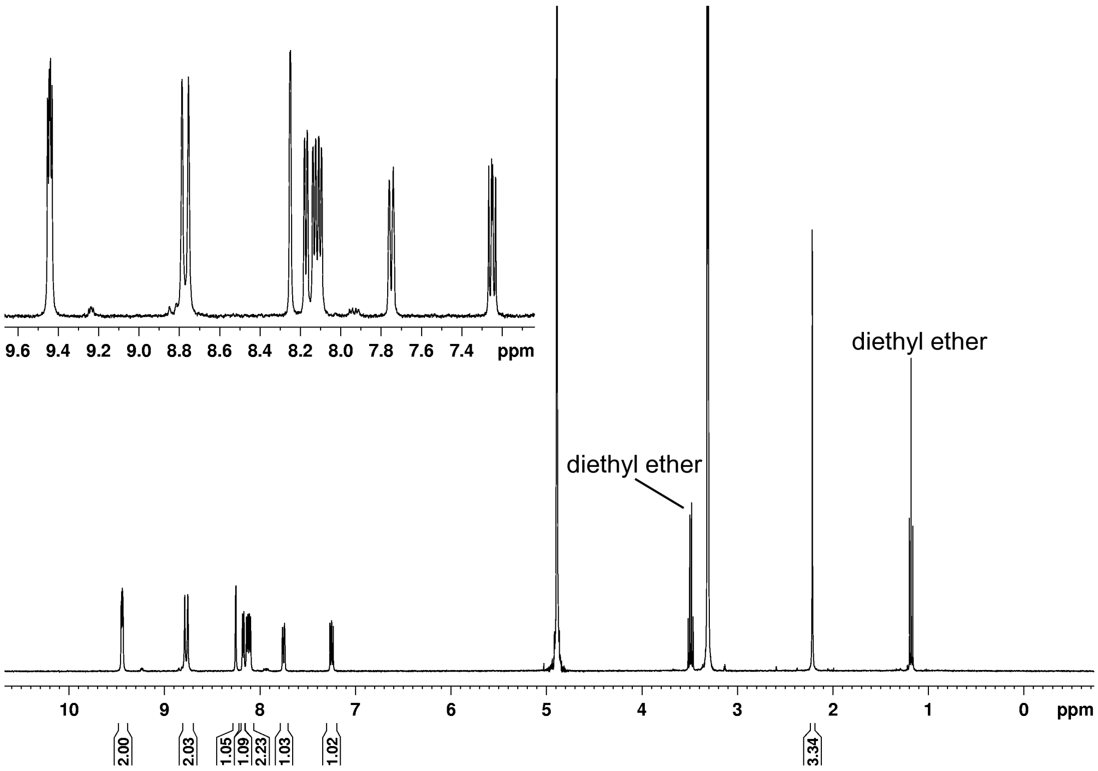

Figure S5. ^{1}H NMR spectrum of ReP^{pic} (CD_3OD, 400 MHz).

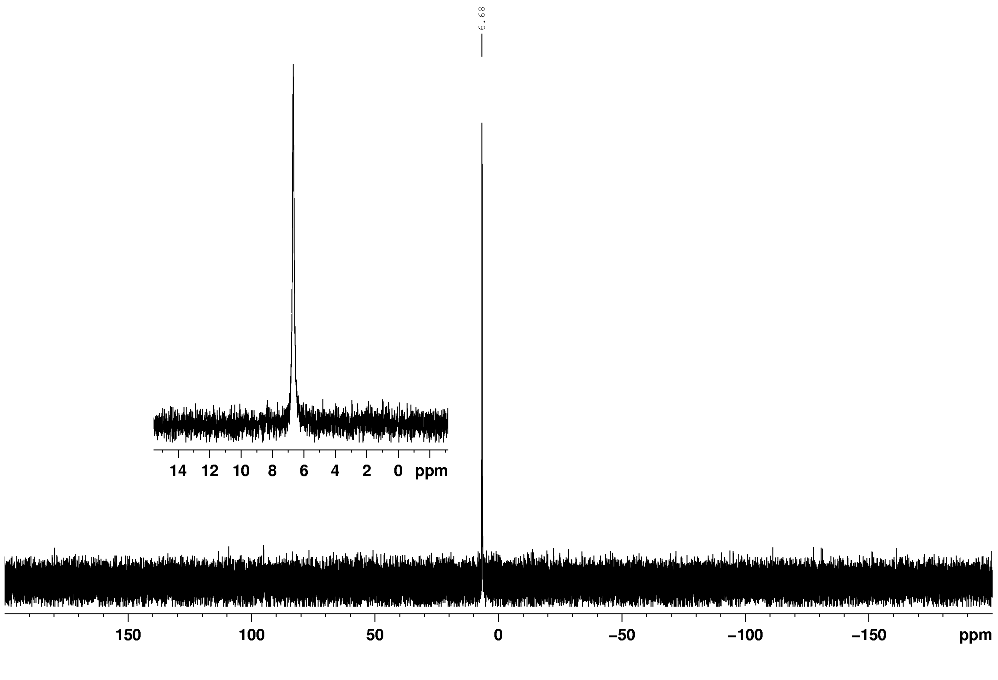

Figure S6. $^{31}P\{^{1}H\}$ NMR spectrum of ReP^{pic} (CD_3OD, 109 MHz).

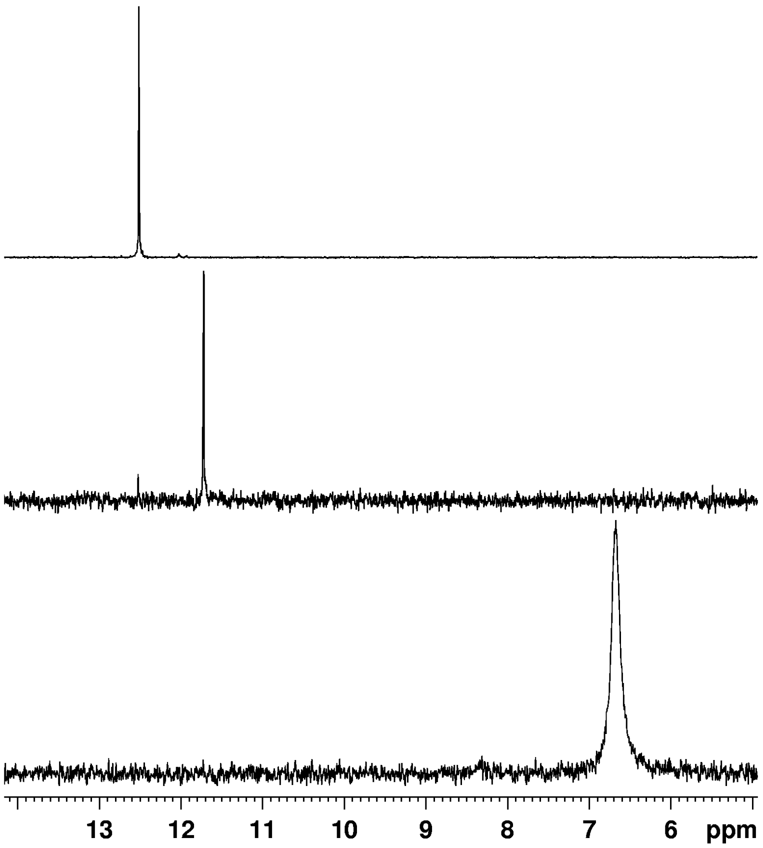

Figure S7. $^{31}P\{^1H\}$ NMR spectra of $^{Et}ReP^{Br}$, $^{Et}ReP^{pic}$ and ReP^{pic}, top to bottom (CD_3OD, 109 MHz).

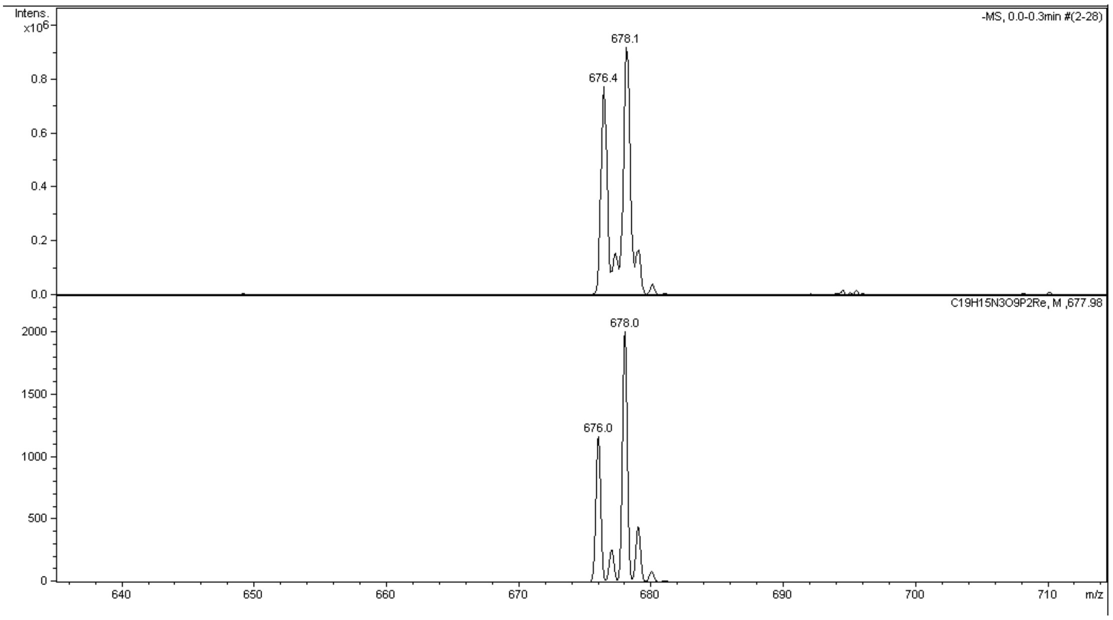

Figure S8. Negative ion mode ESI-MS of ReP^{pic}. Top: experimentally observed spectrum, bottom: calculated spectrum.

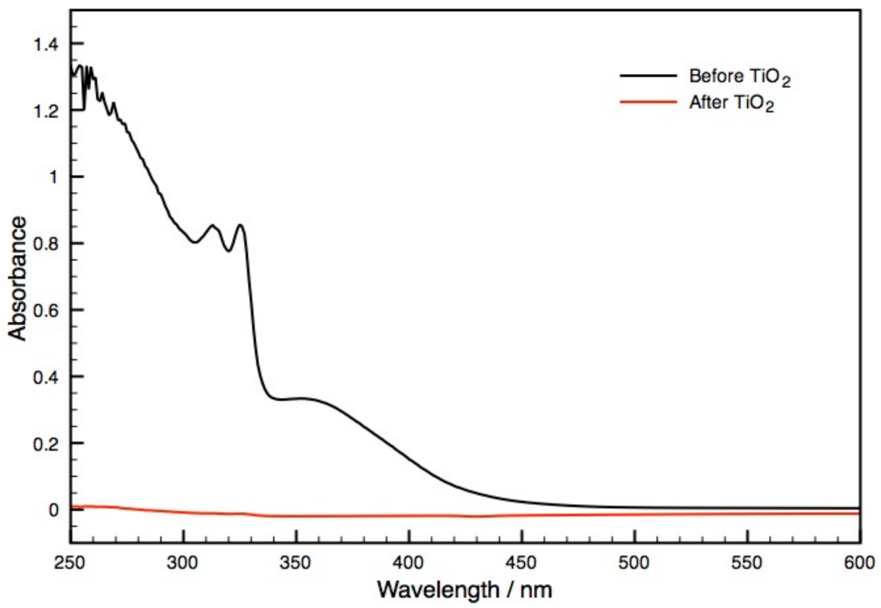

Figure S9. UV-Vis absorption spectra of a solution of ReP^{pic} (0.06 mM in 1.5 mL of 0.1 M TEOA buffer at pH 7) before and after exposure to TiO_2 nanoparticles (5 mg). See main text for further details.

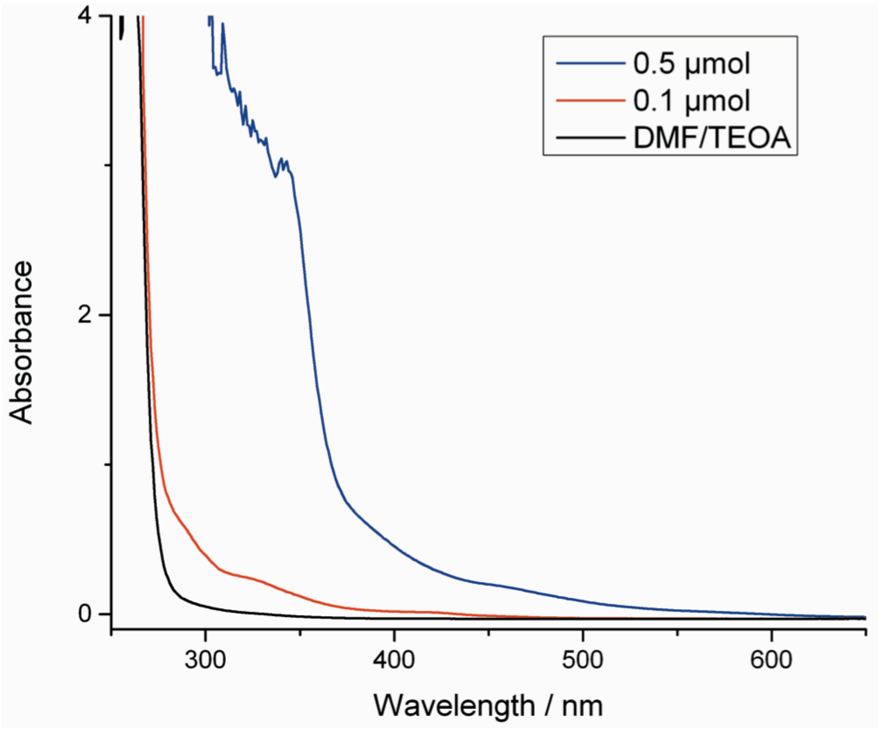

Figure S10. UV-Vis absorption spectra of the supernatant after catalysis for ReP^{pic}-TiO_2 at loadings of 0.1 µmol and 0.5 µmol per 5 mg of TiO_2. See main text for further details.

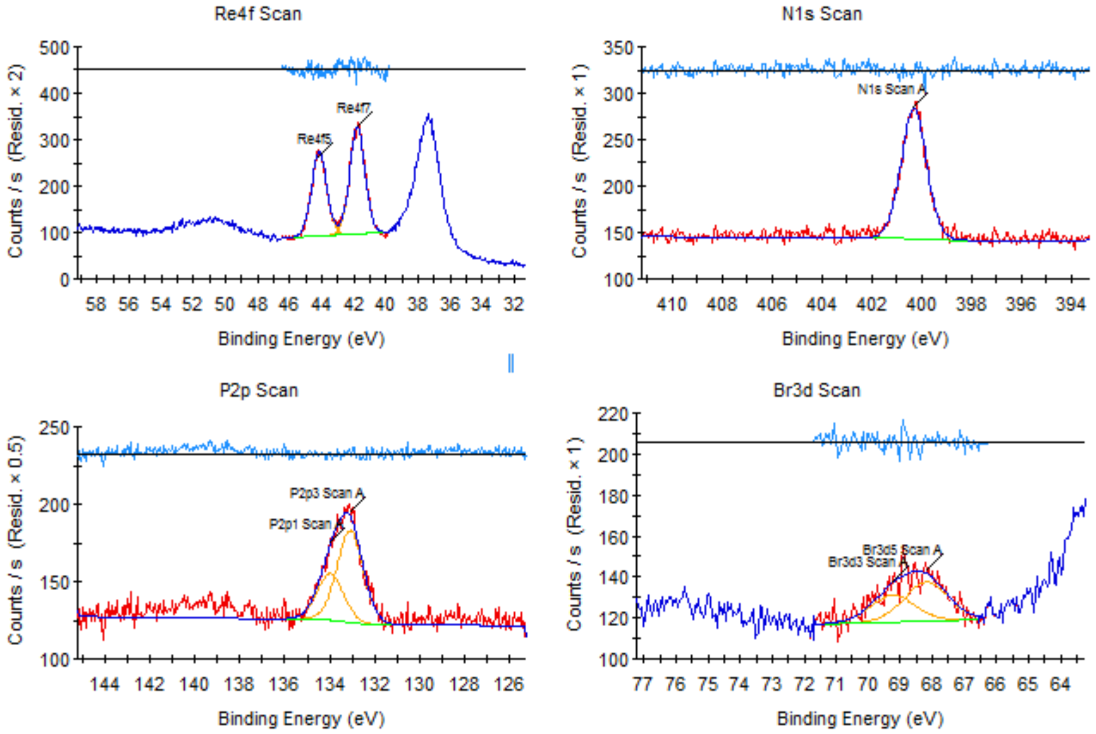

Figure S11. X-ray photoelectron spectra for Re, N, P and Br of ReP^{Br}-TiO_2 before photocatalysis.

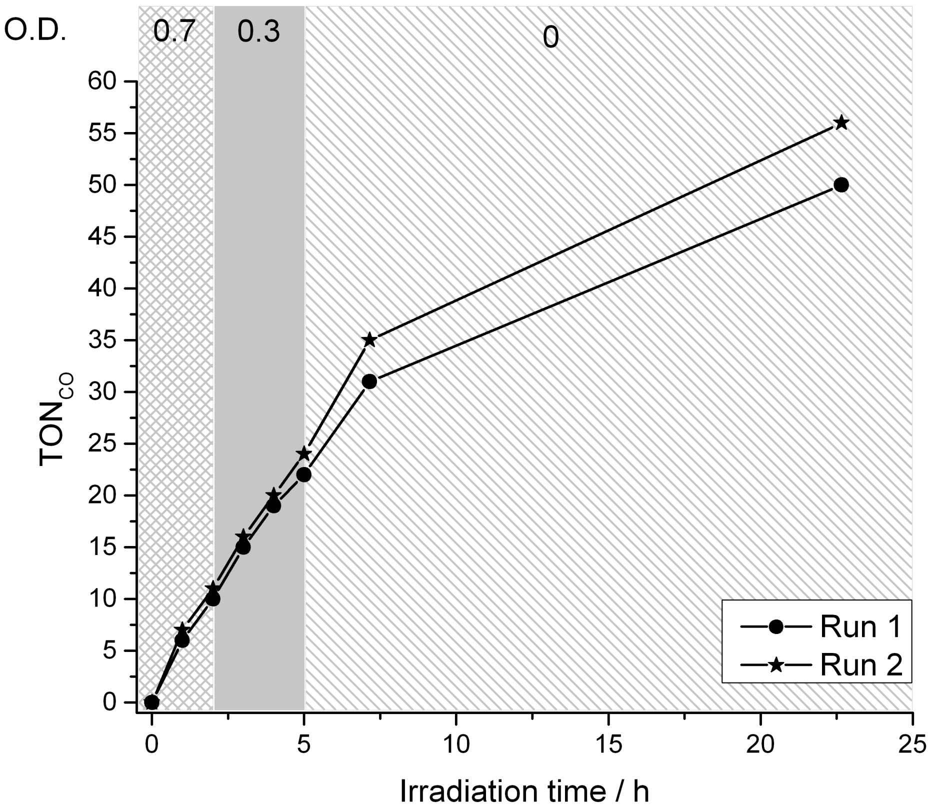

Figure S12. ReP^{pic}-TiO_2 photocatalysis using λ > 420 nm with different neutral density filters (O.D. denotes optical density of the filters).

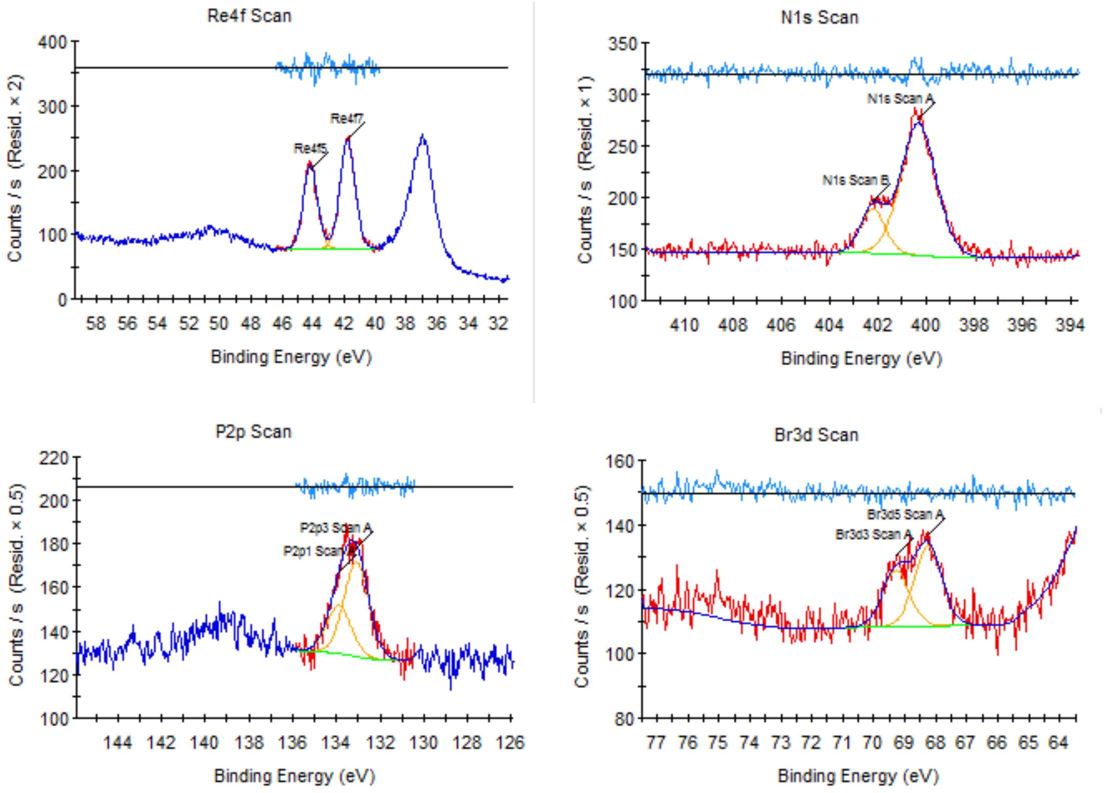

Figure S13. X-ray photoelectron spectra for Re, N, P and Br of ReP^{Br}-TiO_2 after 2 h photocatalysis.

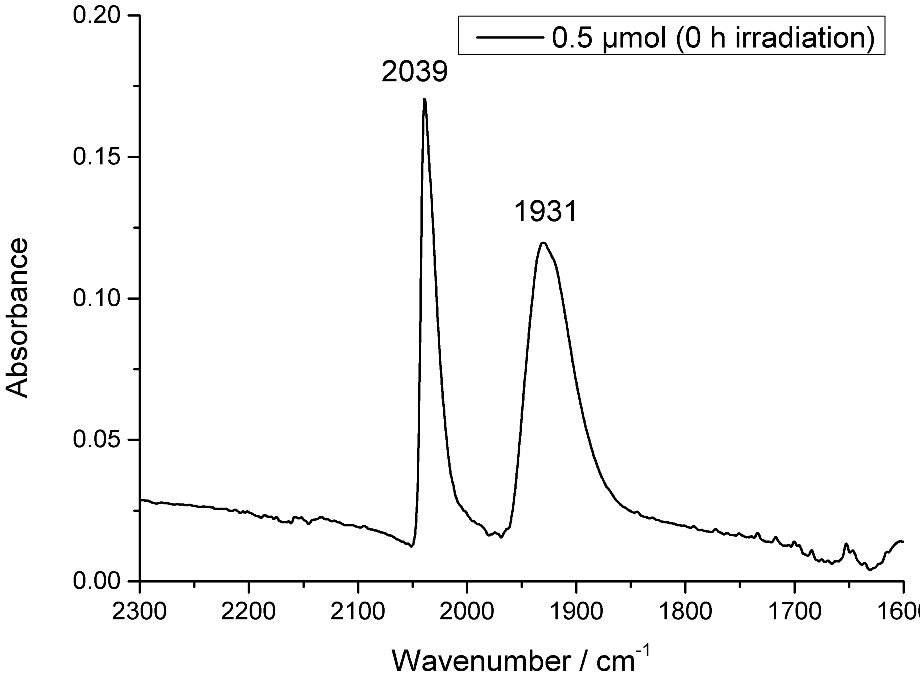

Figure S14. ATR-IR spectrum of ReP^{pic}-TiO_2 before catalysis, taken against a background of TiO_2. Particle loaded at 0.5 µmol ReP^{pic} per 5 mg TiO_2.

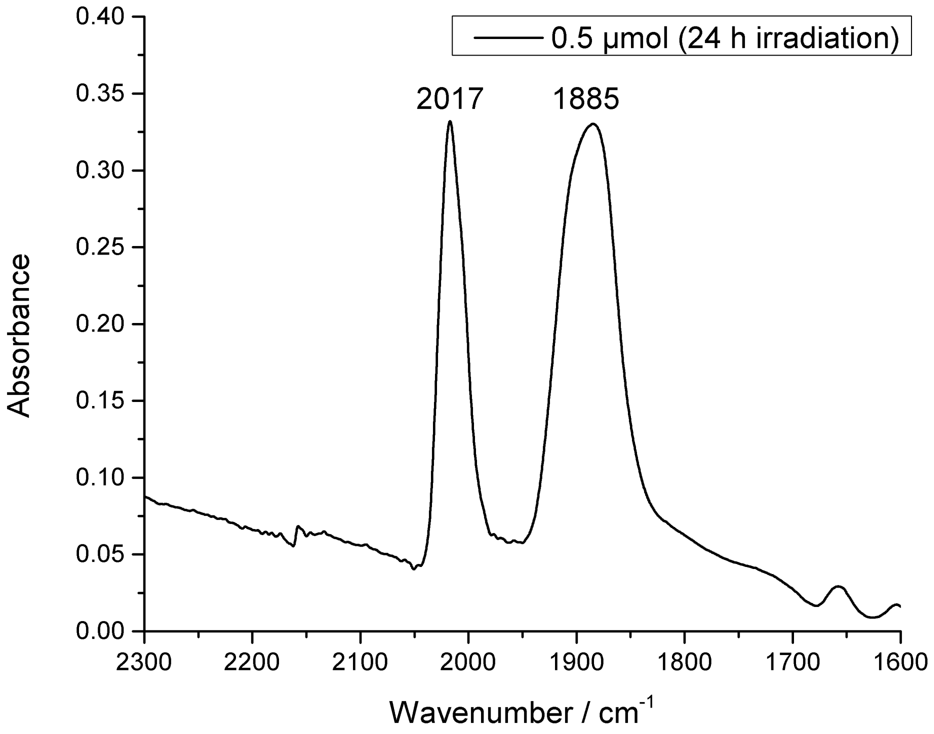

Figure S15. ATR-IR spectrum of ReP^{pic}-TiO_2 after 24 h of photocatalysis, taken against a background of TiO_2 treated with DMF:TEOA in the same way as ReP^{pic}-TiO_2. Particle loaded at 0.5 μmol ReP^{pic} per 5 mg TiO_2.

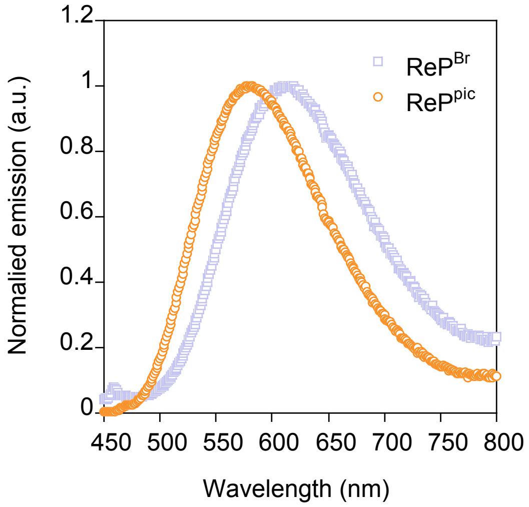

Figure S16. Emission spectra of ReP^{pic} and ReP^{Br} (0.1 mM in water) under N_2 atmosphere with excitation at 404 nm.

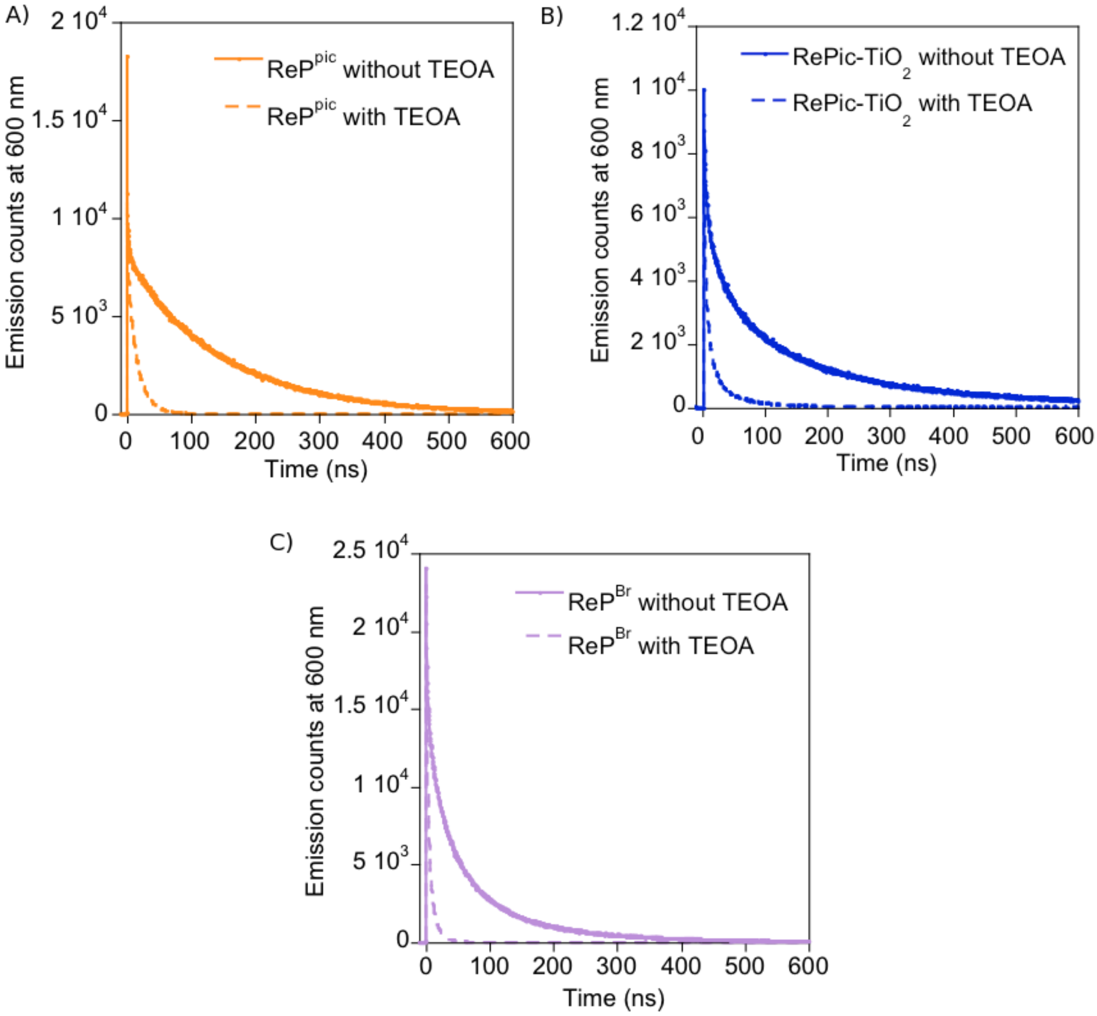

Figure S17. Photoluminescence decays of A) 0.1 mM ReP^{pic} B) ReP^{pic}-TiO_2 and C) ReP^{Br} in water:DMF (1:2), in the absence (solid line) and presence (dashed line) of a sacrificial electron donor TEOA (1 M). The luminescence was monitored at 600 nm with 404 nm excitation.

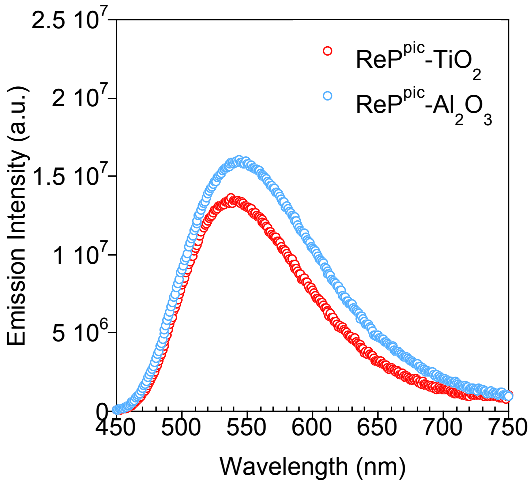

Figure S18. Emission spectra of ReP^{pic} anchored to TiO_2 and Al_2O_3 with excitation at 404 nm. The samples were measured in air.

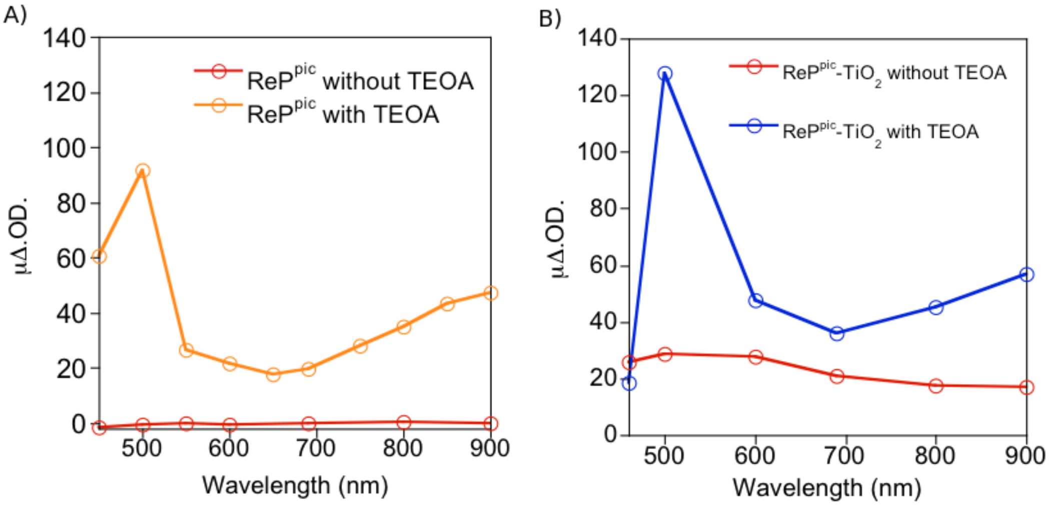

Figure S19. Transient absorption spectrum of A) a 0.1 mM ReP^{pic} solution in DMSO and B) ReP^{pic}-TiO_2 in DMF, measured 300 µs after the excitation laser pulse, with and without sacrificial electron donor (TEOA, 1 M). The samples were excited with visible light (415 nm, ~300 µJ cm^{-2}, 0.5 Hz rep. rate). The amplitude ratio between the peaks at 500 nm and 900 nm was calculated by taking the signal amplitude without TEOA as baseline, in order to account for the background noise at small changes in optical density.

End of Supporting Information
